# CBF-dependent and CBF-independent regulatory pathways contribute to the differences in freezing tolerance and cold-regulated gene expression of two Arabidopsis ecotypes locally adapted to sites in Sweden and Italy

**DOI:** 10.1371/journal.pone.0207723

**Published:** 2018-12-05

**Authors:** Sunchung Park, Sarah J. Gilmour, Rebecca Grumet, Michael F. Thomashow

**Affiliations:** 1 MSU-DOE Plant Research Laboratory, Michigan State University, East Lansing, Michigan, United States of America; 2 Department of Horticulture, Michigan State University, East Lansing, Michigan, United States of America; 3 MSU Plant Resilience Institute, Michigan State University, East Lansing, Michigan, United States of America; 4 Department of Plant, Soil, and Microbial Sciences, Michigan State University, East Lansing, Michigan, United States of America; National Taiwan University, TAIWAN

## Abstract

*Arabidopsis thaliana* (Arabidopsis) increases in freezing tolerance in response to low nonfreezing temperatures, a phenomenon known as cold acclimation. The CBF regulatory pathway, which contributes to cold acclimation, includes three genes—*CBF1*, *CBF2* and *CBF3*—encoding closely-related transcription factors that regulate the expression of more than 100 genes—the CBF regulon—that impart freezing tolerance. Here we compare the CBF pathways of two Arabidopsis ecotypes collected from sites in Sweden (SW) and Italy (IT). Previous studies showed that the SW ecotype was more freezing tolerant than the IT ecotype and that the IT ecotype had a nonfunctional *CBF2* gene. Here we present results establishing that the difference in *CBF2* alleles contributes to the difference in freezing tolerance between the two ecotypes. However, other differences in the CBF pathway as well as CBF-independent pathways contribute the large majority of the difference in freezing tolerance between the two ecotypes. The results also provided evidence that most cold-induced CBF regulon genes in both the SW and IT ecotypes are coregulated by CBF-independent pathways. Additional analysis comparing our results with those published by others examining the Col-0 accession resulted in the identification of 44 CBF regulon genes that were conserved among the three accessions suggesting that they likely have important functions in life at low temperature. The comparison further supported the conclusion that the CBF pathway can account for a large portion of the increase in freezing tolerance that occurs with cold acclimation in a given accession, but that CBF-independent pathways can also make a major contribution.

## Introduction

The CBF pathway has a prominent role in cold acclimation, the process whereby plants increase in freezing tolerance in response to low temperatures [1–3]. In *Arabidopsis thaliana* (hereafter referred to as Arabidopsis), the pathway includes action of three *CBF* genes which are physically linked in tandem array on chromosome 4 and encode closely related AP2/ERF family transcription factors that bind to the CRT/DRE DNA regulatory element present in the promoters of CBF-regulated genes [4–6]. Within minutes of exposing Arabidopsis to low nonfreezing temperatures, *CBF1*, *CBF2* and *CBF3* [5, 7, 8]—also known as *DREB1b*, *DREB1c and DREB1a*, respectively [9]—are induced followed by induction of CBF-targeted cold-regulated (COR) genes, known as the CBF regulon [2, 10]. Expression of the CBF regulon leads to an increase in freezing tolerance as indicated by the findings that overexpression of *CBF1*, *CBF2*, or *CBF3* in transgenic plants results in an increase in freezing tolerance without exposing plants to low temperature [9, 11, 12] and that down-regulation of the CBF pathway in plants exposed to low temperature results in a decrease in freezing tolerance [13, 14].

The natural range of Arabidopsis extends from North Africa to northern Europe and east to Central Asia encompassing geographical locations that differ greatly in winter freezing temperatures [15, 16]. Accordingly, natural variation in freezing tolerance has been observed among Arabidopsis accessions with those collected from cooler northern latitudes generally more freezing tolerant than those collected from warmer southern latitudes [17, 18]. Given the role of the CBF pathway in freezing tolerance, a question that arises is whether natural variation in the pathway has contributed to local adaptation in Arabidopsis. Recent studies provide strong evidence that it has. Ågren and Schemske (2012) [19] conducted reciprocal transplant experiments using Arabidopsis populations collected from sites in Sweden (SW) and Italy (IT) and found that the SW genotype was more fit (measured as survival and total fruit production) than the IT genotype when both genotypes were grown at the Sweden site, and that the reverse was true when both genotypes were grown at the Italy site. Moreover, the relative survival of the IT ecotype at the Sweden site was positively correlated with the minimum soil freezing temperature [19] implicating freezing tolerance as an adaptive trait. Indeed, direct testing showed that the SW ecotype was more freezing tolerant than the IT ecotype and a QTL for both fitness and freezing tolerance was mapped to a site on chromosome 4 that overlapped the *CBF* locus (the QTL was mapped using a RIL population developed from a cross between the IT and SW ecotypes) [20]. These results suggested that differences in the *CBF* locus of the IT and SW ecotypes contribute to the differences in freezing tolerance and the local adaptation observed in the IT and SW ecotypes. Consistent with this suggestion was the finding that the IT ecotype had a nonfunctional *cbf2* gene and that transformation of the SW *CBF2* gene into the IT plants resulted in an increase in freezing tolerance [21].

Here we used CRISPR/Cas9 technology to determine the extent to which the differences in freezing tolerance and cold-regulated gene expression observed between the IT and SW ecotypes are due to differences in their CBF pathways. Our results indicate that the non-functional IT *CBF2* allele contributes to the difference in freezing tolerance between the SW and IT ecotypes, but that other differences in CBF-dependent and CBF-independent function account for the majority of the difference in freezing tolerance between the two ecotypes. Our results also provide evidence that most cold-induced CBF regulon genes in both the SW and IT ecotypes are coregulated by CBF-independent pathways. Finally, we compared our results with those recently published by Zhao et al. (2016) [22] and Jia et al. (2016) [23] who used CRISPR/Cas9 technology to test the role of the CBF pathway in the Col-0 and Col-3 accessions; Col-0 and Col-3 are reported to be derived from the Columbia accession [24, 25]. The comparison identified 44 CBF regulon genes that are conserved among the three accessions, but also revealed that there is considerable diversity in the genes that comprise the IT, Col-0 and SW CBF regulons.

## Materials and methods

### Plant material and growth conditions

The *Arabidopsis thaliana* IT and SW accessions used in this study have been described elsewhere [21]. Seeds were stratified for 3–5 days at 4°C and plants were grown on soil in pots at 22°C under a 12-h or 16-h photoperiod with a light intensity of 100–120 μmol m^−2^ sec^−1^ as described [26]. For RNAseq experiments, the plants were grown on soil in pots for 23 days and then exposed to low temperature (4°C) for 24 h or 2 weeks at about 35 μmol m^−2^ sec^−1^ under a 12-h photoperiod. For evaluation of the vernalization response, the plants were grown on soil in pots at 22°C under a 16-h photoperiod for 12 days and exposed to 4°C under a 16-h photoperiod for 0, 2, 4, 6 and 8 weeks, after which time the plants were moved back to 22°C for flowering.

### Generation of *cbf* mutants using CRISPR/Cas9-mediated genome editing technology

To obtain the mutants with all three CBF proteins being nonfunctional, we utilized the multiplex genome engineering capability of the CRISPR/Cas9 system [27]. A plasmid for CRISPR was obtained from Dr. Jian-Kang Zhu. Briefly, three 19bp oligonucleotides designed to target the coding region of CBF1, CBF2 or CBF3 under control of the AtU3b, AtU6 or At7SL promoter, respectively, were cloned to a single binary vector (pCambia1300): CBF1, 5’-TCGCTGCATTAGCCCTCCG-3’; CBF2, 5’- TCGCCGCCATAGCTCTCCG-3’; CBF3, 5’- GCGGCGGCTGAAGCTGCGT-3’. The binary vector contained three single-guide RNA (sgRNA) modules, each of which was designed to target the coding region of each of the three CBFs. To prevent possible insertional effects by the T-DNA containing the CRISPR/Cas9 transgene, we removed the T-DNA from the CRISPR mutants by backcrossing the homozygous T3 lines of the mutants to the corresponding parents. Independent F2 lines from the backcrosses were used for the experiments to mitigate the formal possibility of off-site mutations affecting the phenotypes of interest.

**Fig 1:**
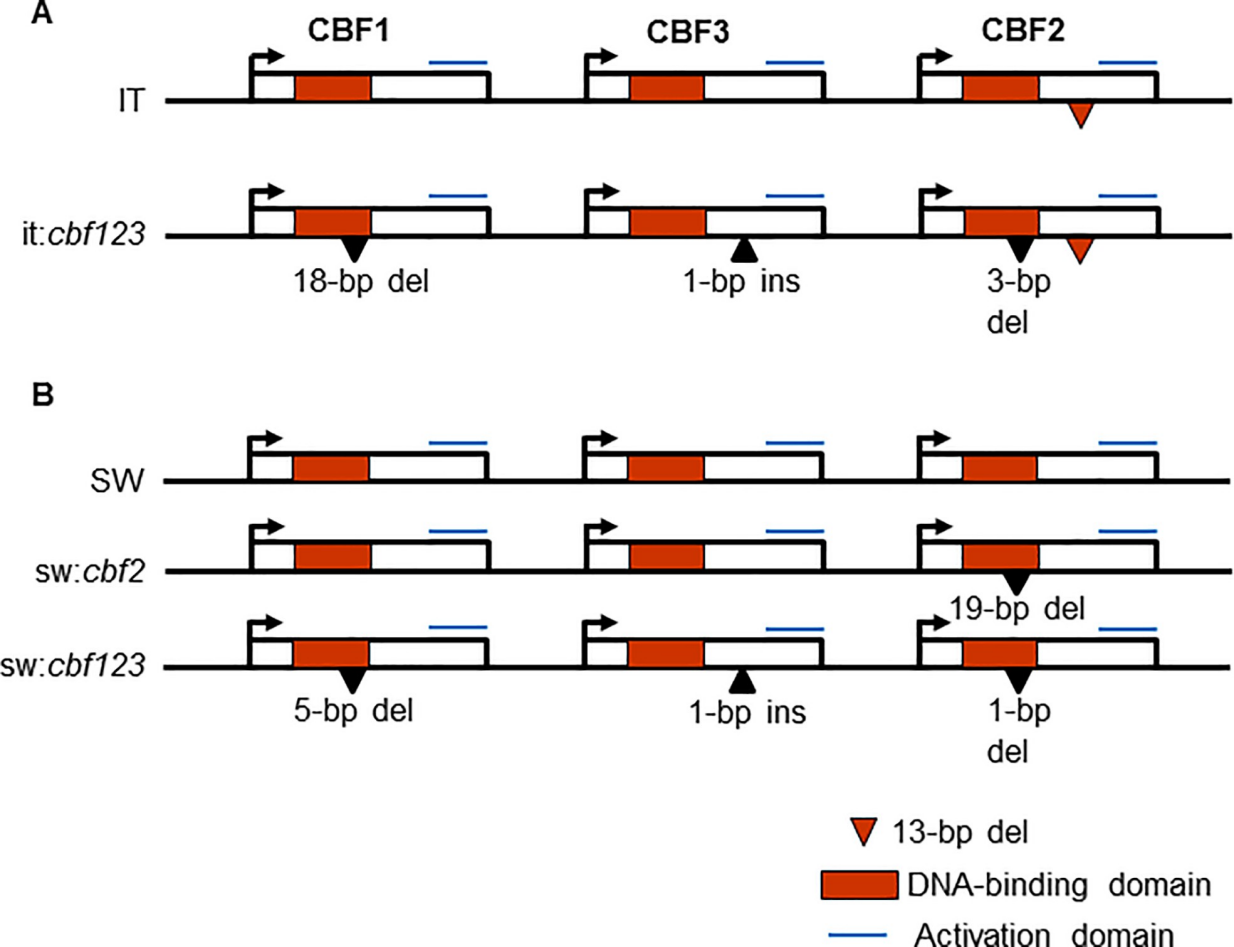
CRISPR-induced mutations in the IT and SW *CBF* genes. Boxes indicate *CBF1*, *CBF2*, and *CBF3* coding regions (red portion indicates DNA binding domain) in the order they are arranged in the genome (not to scale). Black triangles indicate insertion (ins) or deletion (del) mutations. Red triangles indicate site of the natural mutation in the IT *CBF2* gene. The blue lines indicate the activation domain. The mutations within the IT (A) and SW (B) *CBF1*, *CBF2* and *CBF3* coding regions are shown in S1A–S1C Fig.

The IT ecotype has a non-functional *cbf2* allele due to a 13-bp deletion in the C-terminal region of the gene [21]. The frame-shift mutation results in a truncated protein that lacks the C-terminal activation domain (Fig 1; S1B and S1E Fig) and thus, overexpression of the protein does not induce expression of CBF regulon genes [21]. Using the CRISPR/Cas9 system, we made an IT line, it:*cbf123*, that had inactive alleles of all three *CBF* genes. The CRISPR *cbf1* gene had an 18-bp deletion within the DNA binding domain (Fig 1; S1A and S1D Fig). Given that the deletion did not change the reading frame, the modified protein potentially had a functional activation domain and thus might be active. This was not the case as overexpression of the CRISPR cbf1 protein in Ws-2 did not induce expression of CBF regulon genes; in contrast, overexpression of the IT *CBF1* gene did (S2 Fig). The CRISPR *cbf2* gene, which was already inactive had an additional 3-bp deletion in the DNA binding domain (Fig 1; S1B and S1E Fig). The CRISPR *cbf3* gene had a 1-bp mutation predicted to produce a truncated protein that did not have the activation domain (Fig 1; S1C and S1F Fig). Indeed, whereas overexpression of the IT *CBF3* gene induced expression of CBF regulon genes, overexpression of the it:*cbf123* CRISPR *cbf3* gene did not (S2 Fig).

Using the CRISPR/Cas9 system, we also made an SW line with an inactive *cbf2* gene, sw:*cbf2*, and a line that had inactive alleles of all three *CBF* genes, sw:*cbf123* (Fig 1). The *cbf2* gene in sw:*cbf2* had a 19-bp deletion in the DNA binding domain resulting in predicted protein that did not have an activation domain (Fig 1; S1B and S1E Fig). As would be expected, overexpression of this sw:*cbf2 cbf2* gene in Ws-2 did not induce expression of CBF regulon genes, whereas overexpression of the SW *CBF2* gene did (S3 Fig). In the sw:*cbf123* plants, the CRISPR *cbf1* gene had a 5-bp deletion in the DNA binding domain; the *cbf2* gene had a 1-bp deletion in the DNA binding domain; and the *cbf3* gene had a 1-bp deletion upstream of the activation domain (Fig 1; S1A–S1F Fig). None of these proteins had activation domains and thus were presumed to be inactive like the other CBF proteins without activation domains. Indeed, the CRISPR *cbf3* allele in sw:*cbf123* was predicted to form a truncated protein that ended at the same residue as the CRISPR *cbf3* gene in the it:*cbf123* plants, which, as indicated above, produced an inactive protein.

### Generation of transgenic plants overexpressing *CBF alleles*

The coding regions of the *cbf1* allele from it:*cbf123*, the *cbf2* allele from sw:*cbf2*, and the *cbf3* allele from it:*cbf123*, and corresponding WT alleles were amplified by PCR, cloned into the Gateway vector pEarleyGate100 [28] under control of the CaMV 35S promoter, and transformed into Ws-2 plants using the floral dip procedure [29]. Homozygous T3 or higher plants were used for experiments. Primers used for PCR were: CBF1/cbf1, 5’-ACAGAGTACTCTGATCAATGAACTC-3’ (forward) and 5’-GAATATTAGTAACTCCAAAGCGACACG-3 (reverse); CBF2/cbf2, 5’-ATCTTCTACTTACTCTACTCTCA3’ (forward) and 5’-GAATTTTAATAGCTCCATAAGGACACGTCATC-3’ (reverse); CBF3/cbf3, 5’-CAGAGTATTGTTGATCAATGAGCTC-3’ (forward) and 5’-GTTTTAATAACTCCATAACGATACGTCGTCATC-3’ (reverse). The transcript levels for the various transgenes were determined by qRT-PCR (S2 and S3 Figs).

### Quantitative RT-PCR analysis

Total RNA was extracted from plants using RNeasy Plant Mini kits (Qiagen, http://www.qiagen.com) as described [14]. Complementary DNA was synthesized from total RNA with random primers using the Reverse Transcription System (Promega, http://www.promega.com/). Complementary DNA was used as a template for qRT-PCR using fast SYBR Green master mix (Life Technologies) as described [26, 30]. *IPP2* (At3g02780) was used as a reference gene. The primers used for qRT-PCR are shown in S1 Table.

### Freezing tolerance tests

Electrolyte leakage assays were performed as described [30, 31]. Plants were grown at 22°C on soil under a 12-h photoperiod and then transferred to 4°C under a 12-h photoperiod for 2 weeks as described above. EL_50_ values were calculated from fitted third-order linear polynomial trends from the electrolyte leakage curves (S2 Table). Two-way analysis of variance was performed and significance of comparisons of responses among genotypes was determined by orthogonal contrast (S2 Table).

### Promoter motif analysis

Motif enrichment was determined by counting the number of CRT/DRE DNA-binding motifs (rCCGAC) within the region 1 kb upstream of the translation start site for each gene based on TAIR version 10 annotation (Arabidopsis Information Resource; http://www.arabidopsis.org/). Motif enrichment in a group of genes was tested against a background distribution generated by 1000 random samplings from all genes in the Arabidopsis genome and presented as the Z-score and P value.

### RNAseq analysis

Aerial tissue was collected from the IT, SW, CBF2-overexpressing, and *cbf* mutant plants (two experimental replicates) exposed to low temperature (4°C) for 0 h, 24 h and 2 weeks. Total RNA was isolated for each experimental replicate using an RNeasy kit (Qiagen) and submitted to Michigan State University's Research Technology Support Facility (RTSF) for RNAseq library preparation and sequencing. Sequencing was performed on an Illumina Genome Analyzer II (http://www.illumina.com). The RNAseq reads were mapped to the *Arabidopsis thaliana* reference genome (TAIR10) using Bowtie2 version 2.2.6 [32] and Tophat version 2.1.0 [33]. The resulting TopHat BAM files were used to estimate transcript abundance (FPKM) and identify differentially expressed genes using the Cuffdiff program within Cufflinks (version 2.2.1) [33]. Genes with a two-fold change (log_2_ = 1) or more and an FDR = 0.01 were designated as differentially expressed. Hierarchical clustering analyses were performed using the hcluster method of ‘amap’ in the R package (https://www.r-project.org/) and the resulting clusters were visualized with treeview (http://rana.lbl.gov/EisenSoftware.htm). The RNAseq data have been deposited in the Gene Expression Omnibus under accession number GSE106284. Gene ontology term enrichment was performed on the differentially expressed genes for biological processes with the entire nuclear gene set of 33278 (TAIR v10) as a background. P-values were calculated using the hypergeometric test.

## Results

### Differences in *CBF2* alleles contribute to the difference in freezing tolerance between the SW and IT ecotypes

**Fig 2:**
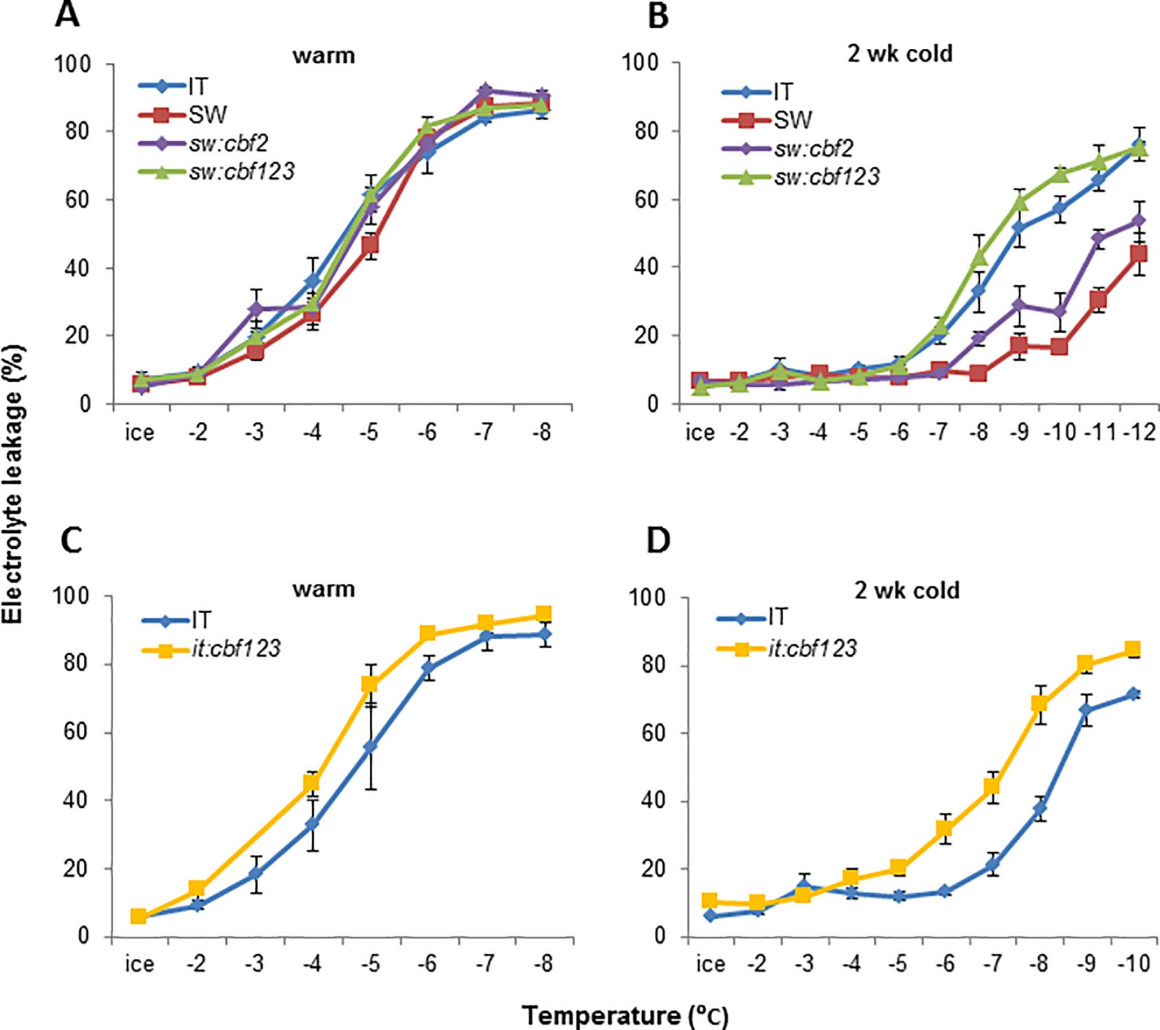
Inactivation of the CBF pathway in SW and IT plants impairs freezing tolerance. Electrolyte leakage freeze tests were conducted on non-acclimated plants grown at 22°C (A, C) and cold-acclimated plants grown at 22°C and then exposed to 4°C for 2 weeks (B, D). sw:*cbf2*, SW with *CBF2* mutated; sw:*cbf123*, SW with *CBF1*, *CBF2*, and *CBF3* mutated; it:*cbf123*, IT with *CBF1*, *CBF2*, and *CBF3* mutated (see Fig 1). Error bars indicate SE of three biological replicates. Third-order linear polynomial trends were fitted for the electrolyte leakage data for each genotype. EL_50_ values were calculated from the fitted models. Two-way analysis of variance was performed; significance of comparisons of responses among genotypes was determined by orthogonal contrast. P values and EL_50_ values are shown in S2 Table.

Oakley et al. (2014) [20] showed that the SW ecotype is more freezing tolerant than the IT ecotype and identified a QTL for freezing tolerance that overlapped the *CBF* locus. Gehan et al. (2015) [21] then showed that the IT *CBF2* gene was nonfunctional and proposed that this accounted for the freezing tolerance QTL that overlapped the *CBF* locus. To test this possibility, we used CRISPR/Cas9 technology to inactivate the SW *CBF2* gene, creating sw:*cbf2* (Fig 1; S1B and S1E Fig; see Materials and Methods) and determined the effects that this had on freezing tolerance (Fig 2; S2 Table). The results indicated that there was little, if any, difference in freezing tolerance between non-acclimated SW and sw:*cbf2* plants (Fig 2A). There was, however, a difference in freezing tolerance between cold-acclimated SW and sw:*cbf2* plants: whereas the EL_50_ (the temperature at which freezing damage results in leakage of 50% of the total cellular electrolytes) of cold-acclimated SW plants was -12.4°C, the EL_50_ of and sw:*cbf2* plants was -11.6°C (Fig 2B; S2 Table). Thus, loss of SW *CBF2* function resulted in a 0.8°C decrease in freezing tolerance indicating that differences in SW and IT *CBF2* alleles did indeed contribute to the difference in freezing tolerance between the two ecotypes.

### Both CBF-dependent and CBF-independent pathways contribute to the freezing tolerance of the SW and IT ecotypes

To determine the extent to which the freezing tolerance of SW and IT plants was dependent on the CBF pathway, we used CRISPR/Cas9 technology to completely inactivate the *CBF* locus of each ecotype—resulting in sw:*cbf123* and it:*cbf123*, respectively (Fig 1; S1A–S1G Fig)—and determined the effects that this had on freezing tolerance (Fig 2; S2 Table). In the case of the SW plants, about 40% of the cold-induced increase in freezing tolerance was CBF-dependent: whereas the freezing tolerance of SW plants increased 7.3°C in response to cold acclimation (EL_50_ values decreased from -5.1 to -12.4°C), the freezing tolerance of sw:*cbf123* plants increased 4.4°C (EL_50_ values decreased from -4.5 to -8.9°C) (Fig 2A and 2B). In contrast, only about 10% of the increase in freezing tolerance that occurred in IT plants in response to low temperature was CBF-dependent: whereas the freezing tolerance of IT plants increased 4.0°C in response to cold acclimation (EL_50_ values decreased from -4.7 to -8.7°C), the freezing tolerance of it:*cbf123* plants increased 3.5°C (EL_50_ values decreased from -3.8 to -7.3°C) (Fig 2C and 2D). In absolute terms, the CBF-dependent pathways of the SW and IT plants contributed, respectively, a 2.9°C and 0.5°C increase in freezing tolerance in response to low temperature whereas the CBF-independent pathways contributed, respectively, a 4.4°C and 3.5°C and increase in freezing tolerance. Thus, both the CBF-dependent and CBF-independent freezing tolerance pathways of the SW plants imparted greater levels of freezing tolerance than did the respective pathways in the IT plants.

### Most cold-induced CBF regulon genes are coregulated by CBF-independent pathways in both the SW and IT ecotypes

**Fig 3:**
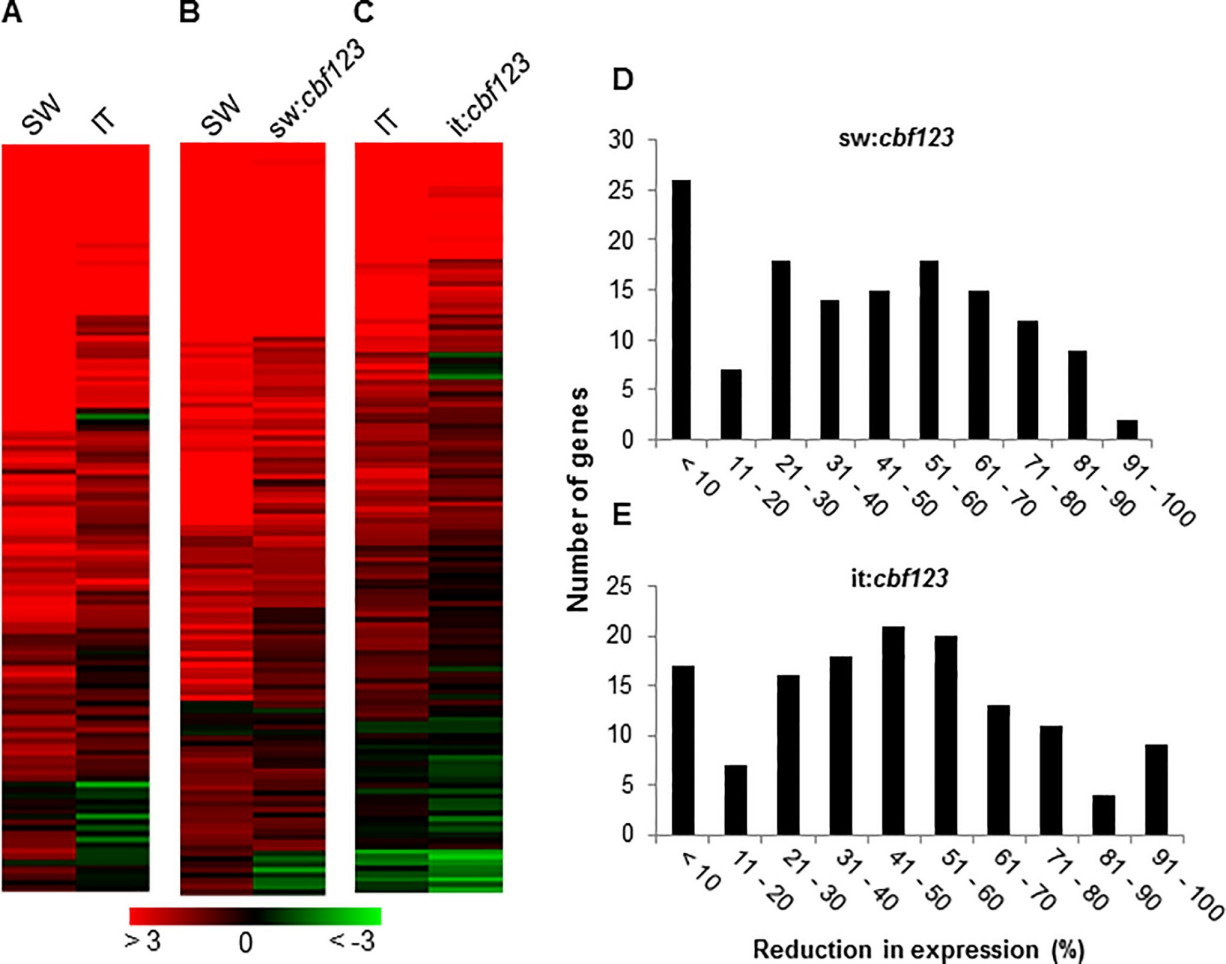
Inactivation of the CBF pathway in the SW and IT ecotypes impairs rapid cold induction of CBF regulon genes. (A, B, C) Transcript levels for the 136 CBF regulon genes of Arabidopsis Ws-2 [14] were compared between (A) IT and SW, (B) SW and sw:*cbf123*, and (C) IT and it:*cbf123* plants after exposure to 4°C for 24 h. Heat maps represent log_2_ fold-change. (D, E) Histograms showing the number of CBF regulon genes that were reduced in expression at low temperature (4°C for 24 h) in the *cbf123* triple mutants compared with their respective WT plants.

Using the Ws-2 accession, Park et al. (2015) [14] identified 136 genes that were induced in plants exposed to low temperature for 24 h and in transgenic plants overexpressing *CBF1*, *CBF2* or *CBF3* grown at warm temperature. They designated these genes members of the CBF regulon. Of these 136 genes, 117 (86%) and 89 (65%) were cold-induced (>2-fold, FDR = 0.01), respectively, in SW and IT plants exposed to low temperature for 24 h (Fig 3A; S3 Table). The transcript levels for most of these CBF regulon genes were reduced in the sw:*cbf123* and it:*cbf123* plants (Fig 3B and 3C; S3 Table), as would be expected for genes induced by the CBF transcription factors. However, a large proportion of these genes were still significantly up-regulated in the sw:*cbf123* and it:*cbf123* plants. Of the 117 CBF regulon genes that were induced in the SW plants, the induction of only 50 genes (43%) was reduced more than 50% in the sw:*cbf123* plants (Fig 3D; S3 Table). Similarly, of the 89 CBF regulon genes that were induced in the IT plants, the induction of only 49 genes (55%) was reduced more than 50% in the it:*cbf123* plants (Fig 3E; S3 Table). These results provide evidence that most of the SW and IT CBF regulon genes were co-regulated by one or more transcription factors in addition to the CBF transcription factors.

**Fig 4:**
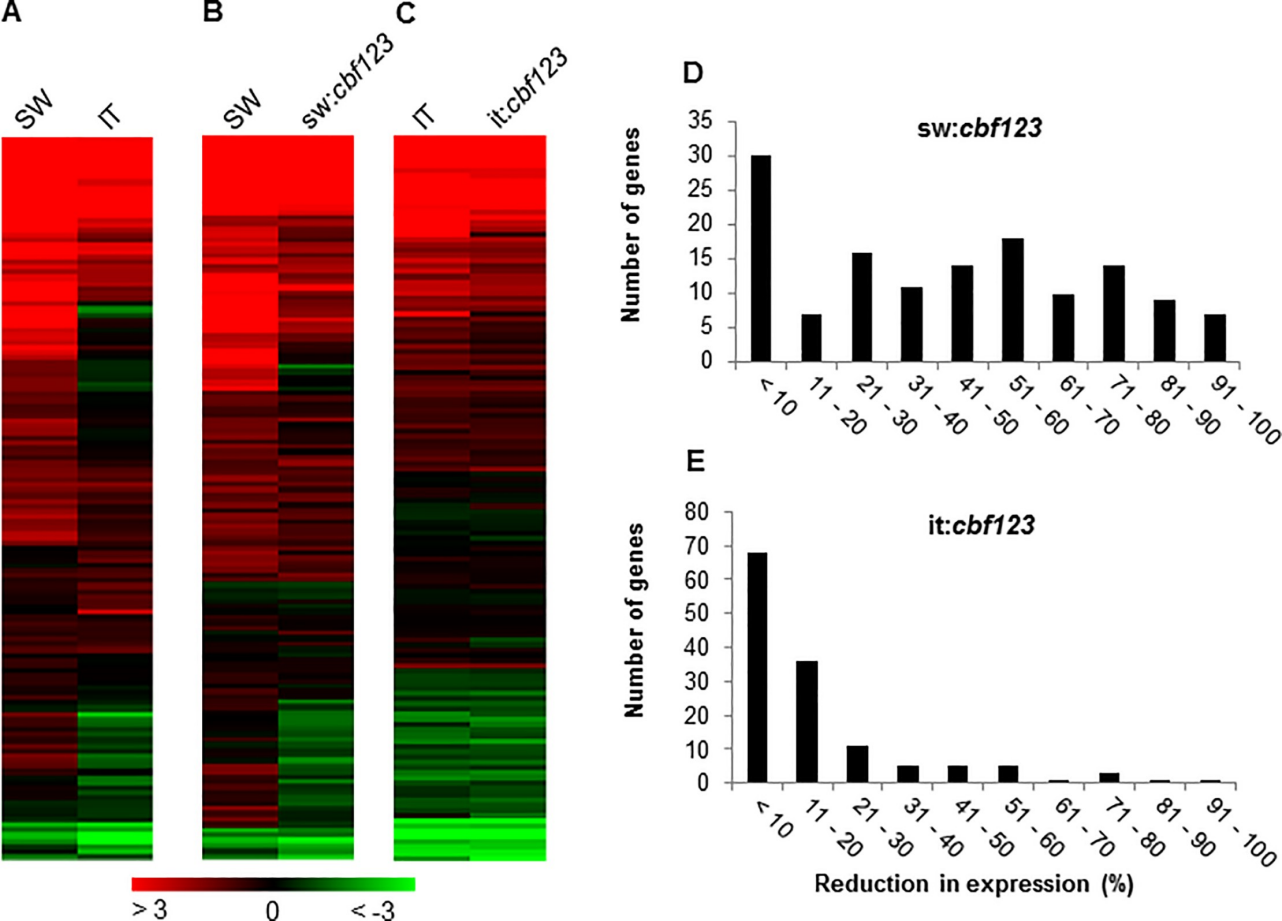
Inactivation of the CBF pathway in the SW and IT ecotypes impairs long-term induction of CBF regulon genes. (A, B, C) Transcript levels for the 136 CBF regulon genes of Arabidopsis Ws-2 [14] were compared between (A) IT and SW, (B) SW and sw:*cbf123*, and (C) IT and it:*cbf123* plants after exposure to 4°C for 2 weeks. Heat maps represent log_2_ fold-change. (D, E) Histogram showing the number of CBF2 regulon genes that were reduced in expression at low temperature (4°C for 2 weeks) in the *cbf123* triple mutants compared with their respective WT plants.

Similar results were obtained when the SW and IT plants were exposed to low temperature for two weeks, though fewer CBF regulon genes were induced at this time point: of the 136 CBF regulon genes, 76 (56%) and 40 (29%) were cold-induced, respectively, in SW and IT plants (Fig 4A; S4 Table). However, there was a striking difference regarding the expression of the CBF regulon genes in the sw:*cbf123* and it:*cbf123* plants. As observed with sw:*cbf123* plants exposed to low temperature for 24 h, the transcript levels for most of the CBF regulon genes were reduced in the sw:*cbf123* plants treated at low temperature for 2 weeks, although again, a large proportion remained significantly up-regulated: of the 76 CBF regulon genes that were induced in the SW plants, the induction of only 39 genes (51%) was reduced more than 50% in the sw:*cbf123* plants (Fig 4B and 4D; S4 Table). In contrast, of the 40 CBF regulon genes that were induced in the IT plants, the induction of only 8 genes (20%) was reduced more than 50% in the it:*cbf123* plants (Fig 4C and 4E; S4 Table). Thus, in the IT genotype, it would appear that most of the CBF regulon genes that were induced after two weeks of cold treatment were up-regulated by transcription factors that were members of CBF-independent pathways.

**Fig 5:**
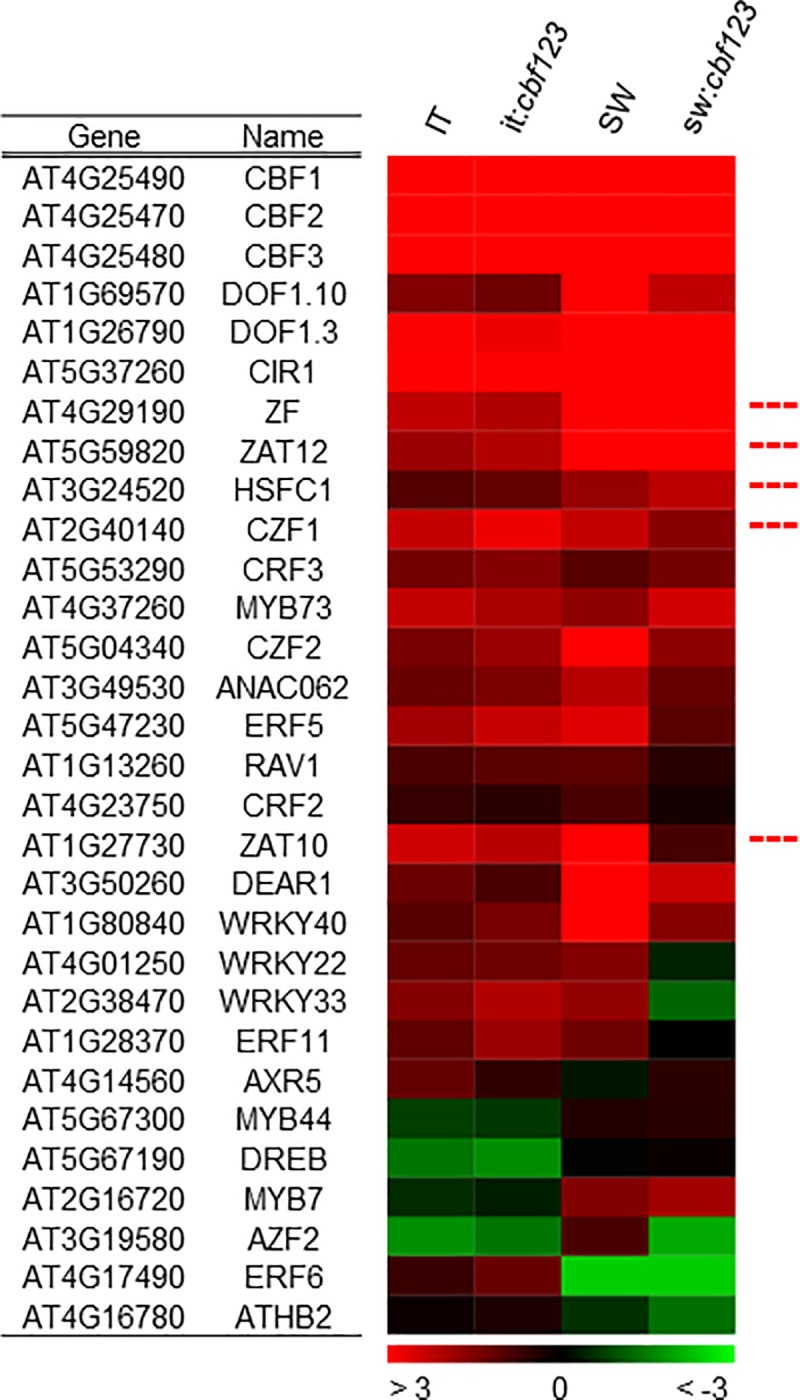
Cold-induced expression of “first-wave” transcription factor genes in SW, IT and their respective *cbf123* triple mutants. Transcript levels of 27 first-wave transcription factor genes of Arabidopsis Col-0 [14] in the IT, SW, and *cbf123* triple mutant plants after exposing plants to 4°C for 24 h. Fold change (log_2_ = 1) in transcript levels is shown comparing transcript levels to those in plants grown at 22°C. The dotted red lines indicate genes encoding transcription factors that have been shown to induce the expression of one or more CBF regulon genes [14].

Park et al. (2015) [14] identified 27 genes in Arabidopsis Col-0 that encoded transcription factors and were rapidly induced in response to low temperature in parallel with *CBF1*, *CBF2* and *CBF3*. Further, they established that five of these “first-wave” genes—*HSFC1*, *ZAT12*, *ZAT10*, *ZF*, and *CZF*—encoded transcription factors that were able to induce one or more of 35 CBF regulon genes. Our results indicated that a majority of the 27 first-wave transcription factor genes of the Col-0 accession—including *HSFC1*, *ZAT12*, *ZAT10*, *ZF*, *CZF*—were also rapidly cold-induced in IT and SW plants indicating that they might also have a role in co-regulating CBF regulon genes in the SW and IT ecotypes (Fig 5). Moreover, cold-induction of *HSFC1*, *ZAT12*, *ZF*, and *CZF* appeared to be independent of the CBF pathway as they were induced in the sw:*cbf123* and it:*cbf123* plants (Fig 5). Thus, induction of CBF regulon genes in the SW and IT ecotypes might also involve co-regulation by a common set of first-wave transcription factors.

### There is considerable diversity in the genes that comprise the CBF regulons of the SW, IT and Col-0 genotypes

**Fig 6:**
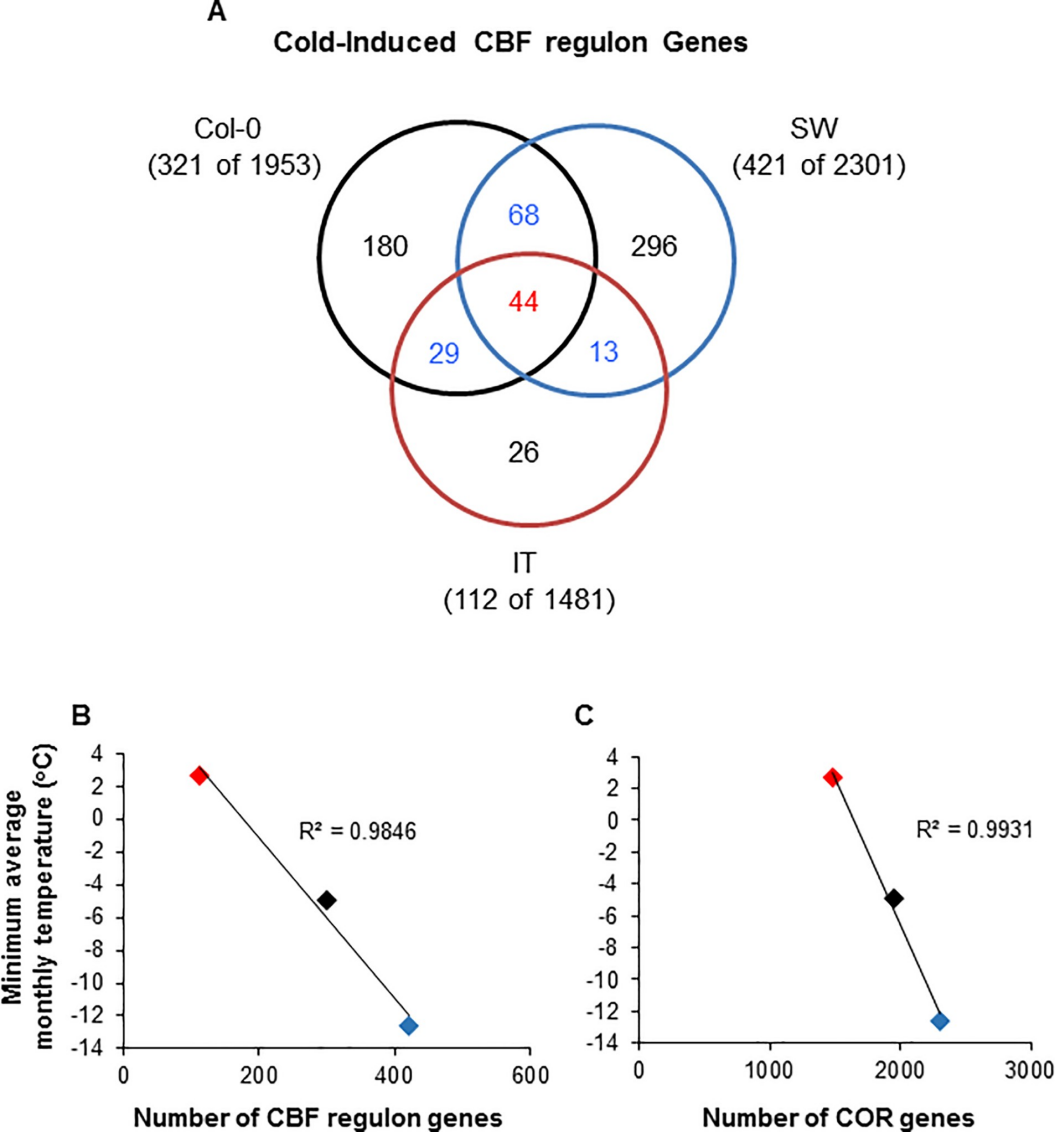
There is considerable diversity in the genes that comprise the CBF regulons of the SW, IT and Col-0 genotypes. (A) Venn diagram showing the number of common and specific CBF regulon genes. Genes were assigned to the CBF regulon if they were induced at least two-fold in response to low temperature (4°C for 24 h) and down-regulated in the *cbf123* triple mutant by at least two-fold (log_2_ = 1; FDR = 0.01 for IT and SW and FDR = 0.05 for Col-0, see text). (B, C) The minimum monthly low temperature average for the genotypes were plotted against the number of CBF regulon genes (B) or total number of COR genes (C). A regression line for the data and R^2^ value are shown. Red indicates it:*cbf123*; black, col:*cbf123*; blue, sw:*cbf123*.

To further explore the issue of CBF regulon diversity, we identified the CBF regulons of the SW and IT ecotypes and compared them with each other and with the CBF regulon of Col-0 determined by Zhao et al (2016) [22]. In this analysis, we defined members of the SW, IT and Col-0 CBF regulons as genes that were cold-induced at 24 h and were reduced at least two-fold in their cold induction in their corresponding *cbf123* triple mutants. The results indicated that there were considerable differences in the number of genes that comprise the CBF regulons of the three genotypes: there were 112, 321 and 421 genes for the IT, Col-0 and SW genotypes, respectively (Fig 6A; S5 and S6 Tables). Moreover, a comparison of the CBF regulon gene lists indicated that there were only 44 genes common to all three genotypes (Fig 6A; Table 1). As would be anticipated, the promoters for these 44 genes in the Col-0 genotype (for which a complete genome sequence is available) were highly enriched in the CBF DNA binding motif, rCCGAC (Table 2). In addition, the motif was highly enriched in the 68 Col-0 CBF regulon genes that were in common with the SW CBF regulon, the 29 Col-0 CBF regulon genes that were in common with the IT CBF regulon, and the 180 Col-0 CBF regulon genes that were “specific” to the Col-0 CBF regulon (Table 2). In contrast, the CBF DNA binding motif was not enriched in the promoters of the 296 CBF regulon genes that were specific to the SW ecotype and the 13 CBF regulon genes that were specific to the SW and IT ecotypes (Table 2), results that were consistent with these genes not being members of the Col-0 CBF regulon.

**Table 1:** CBF regulon genes in common between the SW, IT, and Col-0 genotypes.

NAME	Description	%Reduction sw:*cbf123*/ SW	%Reduction it:*cbf123/* IT	%Reduction col:*cbf123*/ Col
AT2G43620	Chitinase family protein	92.7	83.9	92.9
AT2G16990	Major facilitator superfamily protein	92	62.1	80.1
AT3G22142	Bifunctional inhibitor/lipid-transfer protein	91.8	73.7	66.0
AT4G30830	Protein of unknown function DUF593	91.8	90.5	96.6
AT1G09350	GolS3	90.6	97.1	98.2
AT3G50970	LTI30|dehydrin family protein	88.5	94.9	91.9
AT1G62570	FMO GS-OX4	88.4	87.1	89.6
AT5G52300	RD29B|LTI65|CAP160 protein	85.2	86.1	88.3
AT4G33070	pyruvate decarboxylase family protein	84.8	83.9	61.7
AT4G23450	RING/U-box superfamily protein	83.7	60.9	61.9
AT5G50720	HVA22E|ATHVA22E|HVA22 homologue E	81.8	91.5	75.8
AT5G37500	GORK|gated outwardly-rectifying K+ channel	81.6	71.4	68.3
AT3G55940	phospholipase C family protein	80.7	69.5	92.6
AT3G05660	AtRLP33	79.9	70	95.0
AT4G12470	AZI1|azelaic acid induced 1	77.5	65.4	70.3
AT1G08890	Major facilitator superfamily protein	74.6	82	87.5
AT1G51090	Heavy metal transport/detoxification	74.3	60.6	89.2
AT5G15960	KIN1	74.2	95.1	91.7
AT5G17460	unknown protein	72.8	76.3	84.5
AT1G46768	RAP2.1	71.5	75.5	88.1
AT3G17130	Plant invertase/pectin methylesterase inhibitor	71.5	79.6	86.9
AT3G53990	Adenine nucleotide alpha hydrolases-like	70	53.3	70.0
AT1G47710	Serine protease inhibitor	69.2	52.1	80.0
AT3G62740	BGLU7|beta glucosidase 7	67.3	93.1	67.7
AT1G08920	ESL1, a transporter for monosaccharides	66.9	56.4	59.5
AT1G29395	COR413-TM1	65.8	91.6	87.7
AT5G47650	ATNUDX2|nudix hydrolase homolog 2	62.9	55.9	66.4
AT4G15910	DI21|ATDI21|drought-induced 21	62.8	85	54.2
AT4G21570	Protein of unknown function (DUF300)	62.7	61.9	81.9
AT5G58700	PLC4|phosphatidylinositol-phospholipase C4	60.5	66.4	78.1
AT2G15970	ATCOR413-PM1	58.9	73.6	84.1
AT1G20450	LTI45|ERD10	57.6	62.8	68.2
AT1G64890	Major facilitator superfamily protein	57.5	52.5	83.9
AT4G12000	SNARE associated Golgi protein family	56.4	59.2	79.9
AT5G49900	Beta-glucosidase GBA2 type family protein	56.1	55.6	69.8
AT4G34650	SQS2|squalene synthase 2	55.2	71.9	82.4
AT4G24960	HVA22 homologue D	55	61.4	82.9
AT1G53590	NTMC2T6.1|Calcium-dependent lipid-binding	54.9	54.5	56.7
AT5G10410	ENTH/ANTH/VHS superfamily protein	54.4	57.7	84.4
AT2G42540	cold-regulated 15a	53.7	92.2	95.9
AT5G57110	ACA8|autoinhibited Ca2+ -ATPase isoform 8	53.4	54.6	72.9
AT5G12140	ATCYS1|CYS1|cystatin-1	52.7	62.6	70.0
AT3G09540	Pectin lyase-like superfamily protein	50.9	51.8	57.6
AT2G17280	Phosphoglycerate mutase family protein	50	50.8	70.0

**Table 2:** Enrichment of the CBF-binding motif (rCCGAC).

Class (# of genes)	Z-score	P-value	Occurrence per gene	Expected per gene
Col-0 Specific (180)	10.8	**2E-27**	0.73	0.28
SW specific (296)	1.28	0.101	0.32	0.27
IT specific (26)	2.56	0.005	0.57	0.28
Col-0 and SW (68)	8.21	**1.1E-16**	0.85	0.28
Col-0 and IT (29)	11.03	**1.4E-28**	1.38	0.28
SW and IT (13)	1.25	0.11	0.46	0.27
Common Genes (44)	12.5	**3E-36**	1.34	0.28

In regard to function, the 44 common CBF regulon genes were very highly enriched in the GO terms “response to cold,” “response to water deprivation” and “response to abscisic acid” (Table 3). These three GO terms were also the top very highly enriched terms in the complete CBF regulons of the Col-0 and IT genotypes (Table 3). The CBF regulon of the SW ecotype was also highly enriched in the GO terms “response to water deprivation” and “response to cold”, though not “response to abscisic acid”. Moreover, the SW CBF regulon was very highly enriched in the GO terms related to biotic stress responses including “regulation of plant-type hypersensitive response,” which was not highly enriched in the IT and Col-0 CBF regulons, and “response to chitin” and “defense response to fungus”, which were enriched in the IT and Col-0 CBF regulons, but to a much lesser degree. These results point to the CBF regulons of different genotypes having functional differences beyond the common function of freezing tolerance.

**Table 3:** Enrichment of GO biological processes for CBF regulon genes.

**CBF regulon genes common to the SW, IT and Col-0 genotypes (44)**						
GO ID		GO Term		Target (%)	Genome (%)	P value
GO:0009409		response to cold		36.4	1.2	5.5E-21
GO:0009414		response to water deprivation		34.1	1	1.8E-20
GO:0009737		response to abscisic acid		34.1	1.3	1.4E-18
GO:0009631		cold acclimation		13.6	0.1	2.2E-12
GO:0009269		response to desiccation		11.4	0.1	9.7E-10
**CBF regulon genes of the Col-0 genotype (321)**						
GO ID		GO Term		Target (%)	Genome (%)	P-value
GO:0009409		response to cold		13.1	1.2	9.9E-32
GO:0009737		response to abscisic acid		13.1	1.3	5.5E-30
GO:0009414		response to water deprivation		10.9	1	1.2E-26
GO:0009269		response to desiccation		3.1	0.1	1.2E-12
GO:0009651		response to salt stress		9	1.9	1.7E-12
GO:0042538		hyperosmotic salinity response		4.7	0.5	2.1E-11
GO:0010286		heat acclimation		3.4	0.2	1.5E-10
GO:0006970		response to osmotic stress		3.7	0.4	6.3E-09
GO:0006979		response to oxidative stress		4	0.5	1.2E-08
GO:0009631		cold acclimation		2.2	0.1	1.6E-08
GO:0050832		defense response to fungus		5	0.9	3.5E-08
GO:0010200		response to chitin		5.3	1.3	3.5E-07
GO:0009753		response to jasmonic acid		4	0.8	9.2E-07
GO:0009751		response to salicylic acid		2.8	0.4	5.6E-06
GO:0010310		regulation of hydrogen peroxide metabolic process		3.1	0.5	7.3E-06
GO:0009611		response to wounding		4	1	9.7E-06
**CBF regulon genes of the IT genotype (112)**						
GO ID		GO Term		Target (%)	Genome (%)	P-value
GO:0009409		response to cold		25	1.2	1.1E-30
GO:0009414		response to water deprivation		20.5	1	4.5E-25
GO:0009737		response to abscisic acid		22.3	1.3	4.9E-25
GO:0009631		cold acclimation		6.3	0.1	7.0E-12
GO:0009269		response to desiccation		6.3	0.1	2.0E-11
GO:0009651		response to salt stress		14.3	1.9	8.5E-11
GO:0006970		response to osmotic stress		8	0.4	4.2E-10
GO:0042538		hyperosmotic salinity response		7.1	0.5	3.0E-08
GO:0050832		defense response to fungus		8	0.9	4.6E-07
GO:0009415		response to water		2.7	0	2.2E-06
**CBF regulon genes of the SW genotype (421)**						
GO ID		GO Term		Target (%)	Genome (%)	P-value
GO:0010200		response to chitin		14	1.3	5.6E-44
GO:0009414		response to water deprivation		9.7	1	3.6E-28
GO:0006612		protein targeting to membrane		9.3	1.1	1.0E-24
GO:0010363		regulation of plant-type hypersensitive response		9.3	1.1	1.3E-24
GO:0050832		defense response to fungus		8.6	0.9	6.8E-24
GO:0009867		jasmonic acid mediated signaling pathway		7.8	0.8	1.3E-22
GO:0009611		response to wounding		8.3	1	2.4E-22
GO:0009723		response to ethylene		7.4	0.8	1.4E-21
GO:0009738		abscisic acid-activated signaling pathway		6.9	0.6	1.9E-21
GO:0006865		amino acid transport		5.9	0.4	2.1E-21
GO:0009409		response to cold		8.6	1.2	4.5E-20
GO:0009697		salicylic acid biosynthetic process		5.9	0.6	2.0E-17
GO:0035556		intracellular signal transduction		4.5	0.4	1.2E-14
GO:0030968		endoplasmic reticulum unfolded protein response		5	0.6	2.5E-14
GO:0010310		regulation of hydrogen peroxide metabolic process		4.5	0.5	2.2E-12
GO:0010286		heat acclimation		2.9	0.2	3.1E-10
GO:0009631		cold acclimation		1.4	0.1	3.1E-06
GO:0006979		response to oxidative stress		2.9	0.5	3.2E-06
GO:0015824		proline transport		1.9	0.2	4.2E-06

Finally, we found that there was a highly significant correlation between the number CBF regulon genes in the IT, Col-0 and SW genotypes and the minimum monthly low temperature average of the region from which the genotypes are reported to have been isolated: 2.7°C for IT, -5.0°C for Col-0 and -12.6°C for SW [19, 34] (the Col-0 accession is derived from the La-1 accession which was isolated in Landsberg an der Warthe, now known as Gorzów Wielkopolski, a city in western Poland [24, 25]. Thus, as the minimum monthly low temperature average decreased, the number of CBF regulon genes increased (Fig 6B). A highly significant correlation was also observed between the total number of COR genes and the minimum monthly low temperature average of the region from which the accession was isolated (Fig 6C). It should be noted that our list of CBF regulon and total COR genes was based on using a statistical cut-off of FDR = 0.01 whereas Zhao et al. (2016) [22] used an FDR = 0.05 in their analysis. Using an FDR = 0.05 for analyzing the SW and IT data resulted in a small increase number of CBF regulon genes: SW increased from 421 to 471 and IT increased from 112 to 126 (S4A Fig). However, there was still a highly significant correlation between the number of CBF regulon genes or total COR genes and the minimum monthly low temperature average of the regions from which the accessions were collected (S4B and S4C Fig).

### The CBF pathways of the IT and SW ecotypes retard plant growth at low temperature

**Fig 7:**
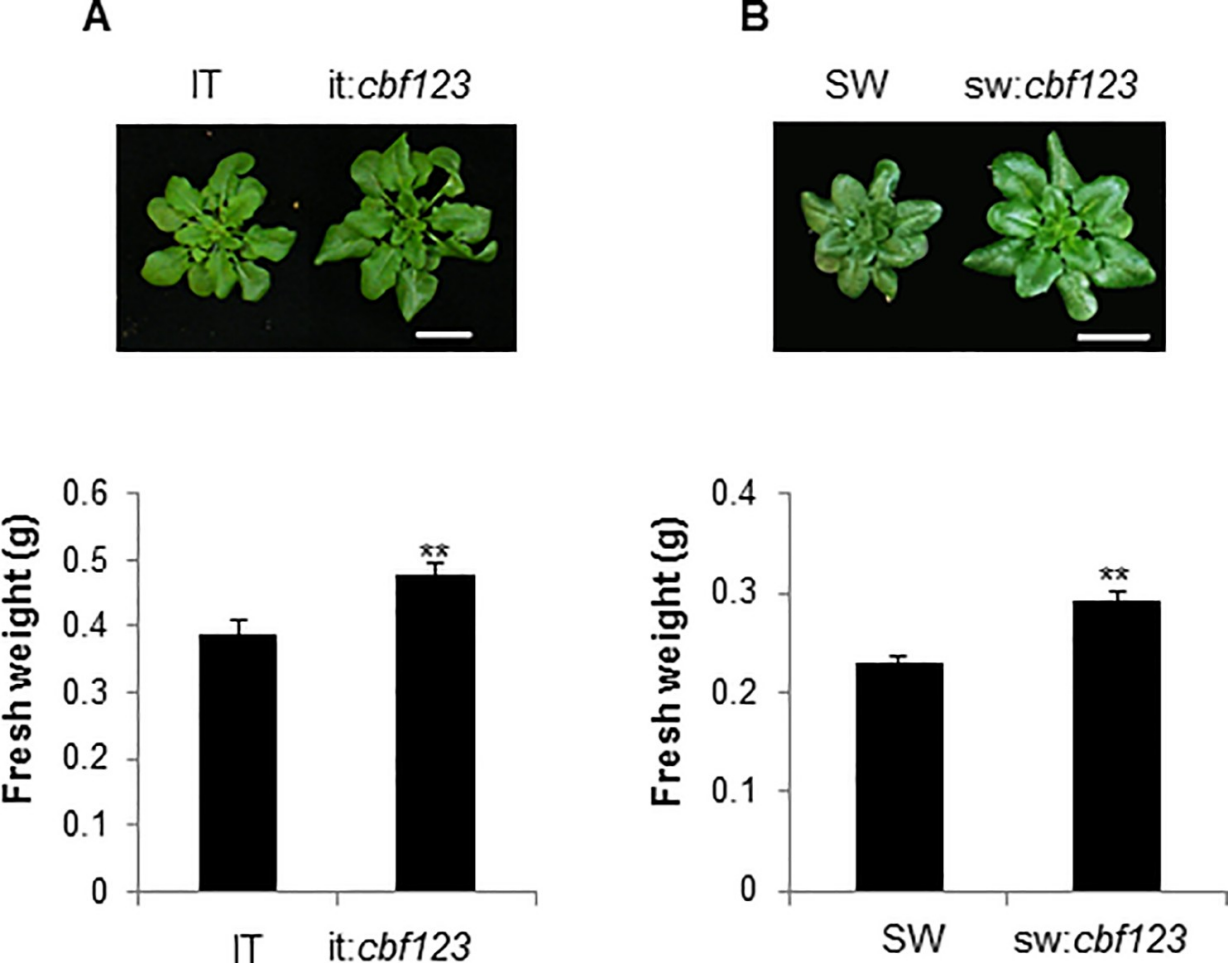
The IT and SW CBF pathways retard plant growth at low temperature. Plants were grown at 4°C under a 16-h photoperiod, and fresh weights of aerial parts were measured after 3 months. (A) IT and it:*cbf123*; (B) SW and sw:*cbf123*. The white bar represents 1 cm. Error bars indicate SE (n = >15 plants). Asterisks indicate a significant difference between WT and the *cbf123* triple mutant (Student t-test, P<0.01).

It is long established that overexpression of the CBF pathway retards plant growth [9, 31, 35]. Consistent with this finding, the fresh weights of IT and SW plants were less than those of it:*cbf123* and sw:*cbf123* plants, respectively, when plants were grown at low temperature for three months (Fig 7).

### Expression of the CBF pathway reduces the time to flowering of IT plants

**Fig 8:**
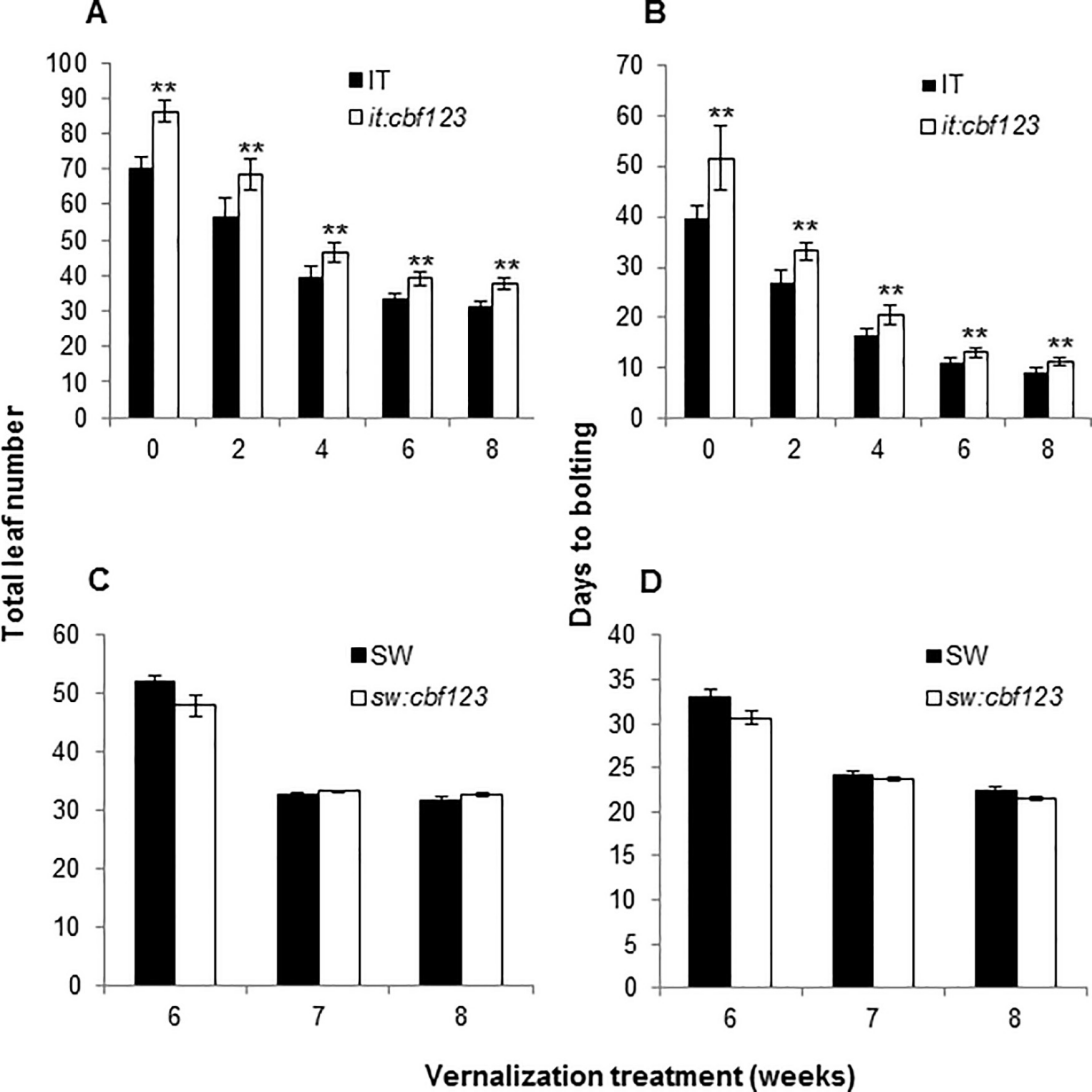
Expression of the CBF pathway reduces the time to flowing of IT plants. Plants were grown at 22°C or 4°C for vernalization under a 16-h photoperiod. (A, B) IT and it:*cbf123*; (C, D) SW and sw:*cbf123*. Flowering time was measured by either total leaf number including rosette and cauline leaves (A, C) or days taken for plants to bolt after transfer to 22°C (B, D). There are no data for SW plants at 22°C (i.e. no 0 weeks vernalization treatment data) because the SW and sw:*cbf123* plants did not flower in the absence of a cold treatment. Error bars indicate SE (n = >9 plants). Asterisks indicate significant differences at each of the indicated time points between WT and mutant (Student t-test, P<0.01).

**Fig 9:**
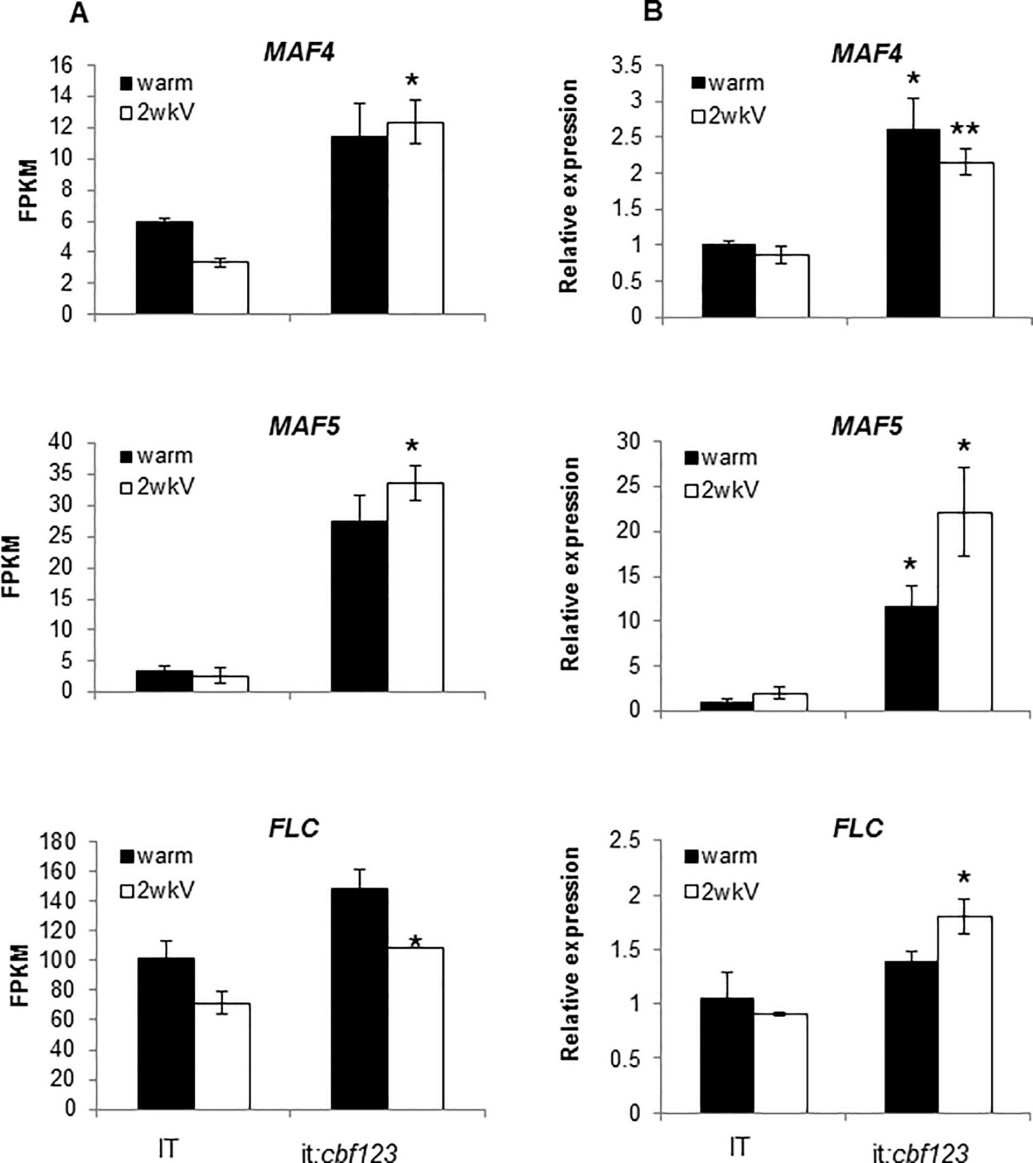
Expression of flowering repression genes *MAF5*, *MAF4* and *FLC* were higher in it:*cbf123* plants than in IT plants. Expression levels of *MAF4*, *MAF5*, and *FLC* were determined either by RNAseq (A) or by quantitative RT-PCR (B) in IT and it:*cbf123* plants grown at 22°C (warm) or at 22°C followed by 2 weeks at 4°C (2wkV) under a 16-h photoperiod. Error bars indicate SE of two (A) or three (B) biological replicates. FPKM = Fragments Per Kilobase of transcript per Million mapped reads. Asterisks indicate a significant difference in expression level between IT and it:*cbf123* plants (Student t-test, * indicates P<0.05, ** indicates P<0.01).

During routine growth of the IT and it:*cbf123* plants at warm temperature, it appeared that the it:*cbf123* plants were generally delayed in flowering. Indeed, quantitative analysis indicated that this was the case: when IT and it:*cbf123* plants were grown at warm temperature, the total number of leaves per generation were 70 and 86, respectively (Fig 8A) and the days to bolting were 40 and 51, respectively (Fig 8B). These results indicated that inactivation of the CBF pathway at warm temperature delayed flowering. Consistent with this observation was our finding that the transcript levels for the flowering repressor genes *MAF5*, *MAF4 and FLC* were higher in it:*cbf123* plants than they were in IT plants (Fig 9A and 9B). An analogous comparison of the SW and sw:*cbf123* plants was not possible as the SW ecotype requires vernalization to flower and thus there could not be a further delay in flowering of plants grown at warm temperature.

### The CBF pathway does not affect the vernalization response of IT or SW plants

Given the effect of the CBF pathway on time to flowering of IT plants, we asked whether the pathway also affected the vernalization response. We found that it did not: the total number of leaves and days to bolting decreased in response to length of vernalization in both the IT and it:*cbf123* plants (Fig 8A and 8B). Similar results were obtained with the SW and sw:*123* plants (Fig 8C and 8D).

## Discussion

One goal of this study was to better understand the genetic basis for the difference in freezing tolerance between the SW and IT ecotypes. Previously, Oakley et al. (2014) [20] used a RIL population developed from a cross between the two ecotypes to map freezing tolerance QTL. Seven loci were identified, one of which overlapped the *CBF* locus with the SW *CBF* locus conditioning greater freezing tolerance than the IT *CBF* locus. Gehan et al. (2015) [21] found that the *CBF2* gene of the IT genotype was nonfunctional and that transformation of the SW *CBF2* gene into the IT plants increased plant freezing tolerance, thus providing indirect evidence that the SW *CBF2* gene contributed to the difference in freezing tolerance between the two ecotypes. Here, we directly tested this hypothesis using CRISPR/Cas9 technology to inactivate the SW *CBF2* gene. The results showed that cold-acclimated SW plants were 0.8°C more freezing tolerant than were cold-acclimated SW plants that had a nonfunctional *cbf2* allele (Fig 2). Thus, the SW *CBF2* gene does indeed contribute to the difference in freezing tolerance between the SW and IT ecotypes.

Although the nonfunctional IT *cbf2* allele contributes to the difference in freezing tolerance between the IT and SW ecotypes, the amount that it contributes would appear to be relatively small; i.e., the increase in freezing tolerance that occurred with cold acclimation of the SW plants was reduced by only about 10% in the sw:*cbf2* plants. Thus, the large majority of the difference in freezing tolerance between the ecotypes would appear to due to differences beyond differences in *CBF2* alleles. In fact, additional differences in the CBF pathways of the IT and SW ecotypes appear to contribute to their difference in freezing tolerance. This suggestion follows from our finding that the CBF pathway accounted for a 2.9°C increase in freezing tolerance in the SW plants, but only a 0.5°C increase in the IT plants, a considerably greater difference than the 0.8°C decrease in freezing tolerance brought about by inactivating the SW *CBF2* gene (Fig 2). One obvious possibility is that either the *CBF1* or *CBF3* gene (or both) of the IT ecotype is not as effective in promoting freezing tolerance as are the corresponding SW *CBF* genes. The results of Oakley et al. (2014) [20] provide some support for this hypothesis. In particular, their genetic analysis indicated that the freezing tolerance QTL overlapping the *CBF2* locus accounted for about 36% of the variance in freezing tolerance between the two ecotypes, considerably more than the 10% difference in freezing tolerance that we observed in converting the SW *CBF2* allele to a nonfunctional *cbf2* allele. It is possible that the differences in the freezing tolerance assays used in the Oakley et al. (2014) [20] study and ours accounts for this difference. Alternatively, it could be due to the single amino acid differences between the SW and IT CBF1 and CBF3 proteins [21] or potentially differences in the level of expression of the genes in general or in specific cell types. These possibilities remain to be tested.

While our results indicate that the IT CBF pathway does not impart the same level of freezing tolerance as does the SW CBF pathway, the results also indicate that there are differences in the level of freezing tolerance promoted by CBF-independent pathways. Whereas CBF-independent pathway genes of the SW plants imparted a 4.4°C increase in freezing tolerance in response to cold acclimation, the CBF-independent pathway genes of the IT plants only imparted a 3.5°C increase in freezing tolerance. In this regard, it is of interest that a number of “first-wave” genes that encode transcription factors are expressed at higher levels in SW plants than they are in IT plants (Fig 5) including two—*ZAT12* and *HSFC1*—that have been shown to impart a small degree of freezing tolerance when overexpressed in the Col-0 accession [14, 36]. Also, eight of the first-wave transcription factors map to the freezing tolerance QTL identified by Oakley et al. (2014) [20]—ZAT10 and ERF11, FrzT1:1; Dof1.10, FrzT1:2; WRKY33 and CZF1, FrzT2:2; CRF2, FrzT4:1; DEAR1 and ATMYB44, FrzT5:2—and thus potentially contribute to the difference in freezing tolerance between the SW and IT ecotypes.

A second goal of this study was to better understand the extent to which the CBF pathway contributes to freezing tolerance in Arabidopsis. Park et al. (2015) [14] addressed this question using a dominant negative version of the CBF2 protein to impair function of the CBF pathway in the Ws-2 accession and found that it accounted for about 50% of the increase in freezing tolerance associated with cold acclimation. Using CRISPR/Cas9 technology, Zhao et al. (2016) [22] compared the freezing tolerance of Col-0 and col-0:*cbf123* plants and found that the CBF pathway accounted for about 60% of the increase in freezing tolerance that occurred with cold acclimation. Similarly, Jia et al. (2016) [23] compared the freezing tolerance of Col-3 and col-3:*cbf123* plants using CRISPR/Cas9 technology—the Col-0 and Col-3 accessions are reported to be derived from the Columbia accession [24]—and obtained results that were consistent with those of Zhao et al. (2016) [22]. And finally, here we used CRISPR/Cas9 technology to create the sw:*cbf123* triple mutant and found that the CBF pathway accounted for about 40% of the increase in freezing tolerance that occurred with cold acclimation in the SW ecotype. Thus, with certain Arabidopsis accessions, the CBF pathway can account for a major portion of the increase in freezing tolerance that occurs with cold acclimation. However, it is also clear that this is not always the case as we observed that the CBF pathway of the IT ecotype only contributed about 15% of the increase in freezing tolerance that occurred with cold acclimation.

While it is clear that the CBF pathway can contribute a large portion of the increase in freezing tolerance that occurs with cold acclimation, it is equally evident that CBF-independent pathways can also contribute significantly to freezing tolerance. At present, knowledge of such pathways is limited. As alluded to above, overexpression of *ZAT12* and *HSFA1*, which encode transcription factors that are rapidly induced in parallel with the *CBF* genes, has been shown to cause an increase in Arabidopsis freezing tolerance [14, 36], though the effects are much less than those caused by overexpression of either CBF1, CBF2 or CBF3 [9, 11]. The CAMTA1, CAMTA2 and CAMTA3 transcription factors have also been shown to contribute significantly to freezing tolerance; about 50% of the increase in freezing tolerance that occurs in Arabidopsis Col-0 plants in response to low temperature [30, 37] involve the CAMTA proteins. However, the CAMTA proteins are positive regulators of *CBF1*, *CBF2* and *CBF3* and thus, it is unclear to what extent the effects that the CAMTA transcription factors have on freezing tolerance are independent of their regulation of the CBF pathway. In short, additional study will be required to better understand the nature of the CBF-independent pathways that contribute as much as half the increase in freezing tolerance that occurs with cold acclimation in the SW, IT, Col-0 and Ws-2 (and likely other) accessions.

A third goal of this study was to better understand the degree to which the composition of the CBF pathway is conserved among Arabidopsis accessions. What we found in comparing our results with those of Zhao et al. (2016) [22] is that the CBF regulons of the SW, IT and Col-0 genotypes have 44 genes in common (of course, this number would increase somewhat if the fold-increase and statistical cutoffs used were less strict). The conserved set of 44 genes was highly enriched for the GO terms “response to cold,” “response to water deprivation” and “response to abscisic acid” (Table 3) which reflects the fact that freezing tolerance must include tolerance to the severe dehydration stress associated with freezing [3, 38]. Indeed, among the 44 conserved CBF regulon genes was *GolS3*, which encodes a key enzyme in the biosynthesis of raffinose, a sugar with cryoprotective and osmoprotective activities [39–41], and multiple LEA (and LEA-like) proteins, including COR15A, LTI45, KIN1 and LTI65, which are thought to protect proteins and membranes against damage caused by severe dehydration [42, 43]. Presumably, the other conserved CBF regulon genes also have important roles in life at low temperature, though the nature of these roles remains to be determined.

In addition to identifying a core set of conserved CBF regulon genes, our results combined with those of Zhao et al. (2016) [22] indicate that CBF regulons can differ greatly in gene number: the CBF regulons of the IT, Col-0 and SW genotypes were composed of 112, 321 and 421 genes, respectively. These differences would seem to have biological significance as the number CBF regulon genes in the three genotypes highly correlated with the minimum monthly low temperature average of the region from which the genotypes were isolated (Fig 6B; S4B Fig): as the minimum monthly low temperature average decreased, the number of CBF regulon genes increased. This correlation suggests that those plants which experience severe cold temperatures might require specific gene functions that are not required by plants which inhabit environments with more moderate low temperatures. The same suggestion applies to genes outside the CBF regulon as the total number of COR genes also varied greatly between the IT, Col-0 and SW genotypes and there was a significant correlation of this number with the minimum monthly low temperature average of the environment (Fig 6C; S4C Fig). These results add to the previous work of Hannah et al. (2006) [44] who reported a correlation between the freezing tolerance of nine Arabidopsis accessions and the number of genes that were differentially expressed in response to low temperature.

Finally, our results suggest that CBF pathways may have evolved to include differences in gene composition that contribute to local adaptation beyond conditioning the core function of freezing tolerance. In particular, the SW CBF regulon was found to be very highly enriched for genes that comprise the GO categories “regulation of plant-type hypersensitive response” and “jasmonic acid mediated signaling pathway,” categories that are not highly enriched in either the IT or Col-0 genotypes (Table 3). We also found that inactivation of the CBF pathway delayed flowering in the IT ecotype, but not the SW ecotype, a difference that might contribute to IT fitness in its natural habitat. The ability to use gene editing technologies to inactive the *CBF1*-*CBF2*-*CBF3* locus will facilitate studies to better understand the evolution of CBF regulon functions that have contributed to local adaptation of Arabidopsis.

## Supporting information

S1 Fig Alignment of nucleotide and amino acid sequences for the CBF coding regions in SW, IT and CRISPR mutant plants. Nucleotide sequences for *CBF1* (A), *CBF2* (B) and *CBF3* (C) and amino acid sequences for CBF1 (D), CBF2 (E) and CBF3 (F) in IT, it:c*bf123*, SW, sw:*cbf2* and sw:*cbf123* plants. Differences between IT and SW sequences are indicated in red; CRISPR-induced mutations are indicated in blue. In protein sequences, red and blue lines indicate the AP2 DNA-Binding Domain and Activation Domain, respectively.
(PPTX)

S2 Fig The *cbf1* and *cbf3* alleles from it:*cbf123* plants encode non-functional proteins. The *cbf1* and *cbf3* alleles and corresponding *CBF1* and *CBF3* WT coding sequences from the IT ecotype were overexpressed in Ws-2 plants to determine whether they could induce expression of CBF regulon genes. (A) Photographs show that overexpression of the *cbf1* and *cbf3* alleles did not retard plant growth consistent with the proteins being non-functional. (B) Overexpression of *cbf1* and *cbf3* did not induce expression of the CBF regulon genes *Gols3*, *COR15a* or *COR47*. Names in parentheses on the x-axis indicate the lines from which the overexpressed alleles are cloned. Error bars indicate SE for three biological replicates.
(PPTX)

S3 Fig The *cbf2* allele from sw:*cbf2* plants encodes a non-functional protein. The *cbf2* allele and corresponding *CBF2* coding sequence from the SW ecotype were overexpressed in Ws-2 plants to determine whether they could induce expression of CBF regulon genes. (A) Photographs show that overexpression of the *cbf2* allele did not retard plant growth consistent with the protein being non-functional. (B) Overexpression of *cbf2* did not induce expression of the CBF regulon genes *Gols3*, *COR15a* or *COR47*. Names in parentheses on the x-axis indicate the lines from which the overexpressed alleles are cloned. Error bars indicate SE for three biological replicates.
(PPTX)

S4 Fig The CBF regulons of the IT, SW, and Col-0 genotypes are composed of conserved and specific genes. (A) Venn diagram showing the number of common and specific CBF regulon genes. Genes were assigned to the CBF regulon if they were induced at least two-fold in response to low temperature (4°C for 24 h) and down-regulated in the *cbf123* triple mutant by at least two-fold (log_2_ = 1, FDR = 0.05). (B, C) The average minimum monthly temperatures for the genotypes were plotted against the number of CBF regulon genes (B) or total number of COR genes (C). A regression line for the data and R^2^ value are shown. Red indicates it:*cbf123*; black, col:*cbf123*; blue, sw:*cbf123*.
(PPTX)

S1 Table Primers used for quantitative RT-PCR. (DOCX)

S2 Table EL_50_ values and statistical analysis of temperature response curves. EL_50_ values were calculated from fitted third-order linear polynomial trends from the curves shown in Fig 2 and are shown in the diagonal boxes. Two-way analysis of variance was performed for the electrolyte leakage experiment; significance of comparisons of responses among genotypes was determined by orthogonal contrast. P values representing comparisons of the temperature response curves are indicated in the intersecting cells.
ns = not significantly different
Comparison of each genotype in both Fig 2A & 2B and in Fig 2C & 2D indicated the curves were very highly significantly different (P<0.0001) between warm and 2 wk cold.
(DOCX)

S3 Table Transcript levels for the 136 Ws-2 CBF regulon genes [14] in SW, sw:*cbf123*, IT and it:*cbf123* plants after a 24 h cold treatment. (XLSX)

S4 Table Transcript levels for the 136 Ws-2 CBF regulon genes [14] in SW, sw:cbf*123*, IT and it:*cbf123* plants after a 2 week cold treatment. (XLSX)

S5 Table CBF regulon genes of SW, IT and Col-0 plants shown in the Venn diagram presented in Fig 6A. (XLSX)

S6 Table Expression of the 44 common CBF regulon genes in SW, sw:*cbf123*, IT, it:*cbf123*, Col-0, col-0:*cbf123* plants exposed to low temperature for 24 h. (XLSX)
