# Supplementary material for: CBF-dependent and CBF-independent regulatory pathways contribute to the differences in freezing tolerance and cold-regulated gene expression of two Arabidopsis ecotypes locally adapted to sites in Sweden and Italy
Source: PLoS One. 2018 Dec 5;13(12):e0207723. doi: 10.1371/journal.pone.0207723 (PMC6281195; doi:10.1371/journal.pone.0207723)
Supplement: S1 Fig — Nucleotide sequences for CBF1 (A), CBF2 (B) and CBF3 (C) and amino acid sequences for CBF1 (D), CBF2 (E) and CBF3 (F) in IT, it:cbf123, SW, sw:cbf2 and sw:cbf123 plants. Differences between IT and SW sequences are indicated in red; CRISPR-induced mutations are indicated in blue. In protein sequences, red and blue lines indicate the AP2 DNA-Binding Domain and Activation Domain, respectively. (PPTX) [file pone.0207723.s001.pptx]

## Slide 1
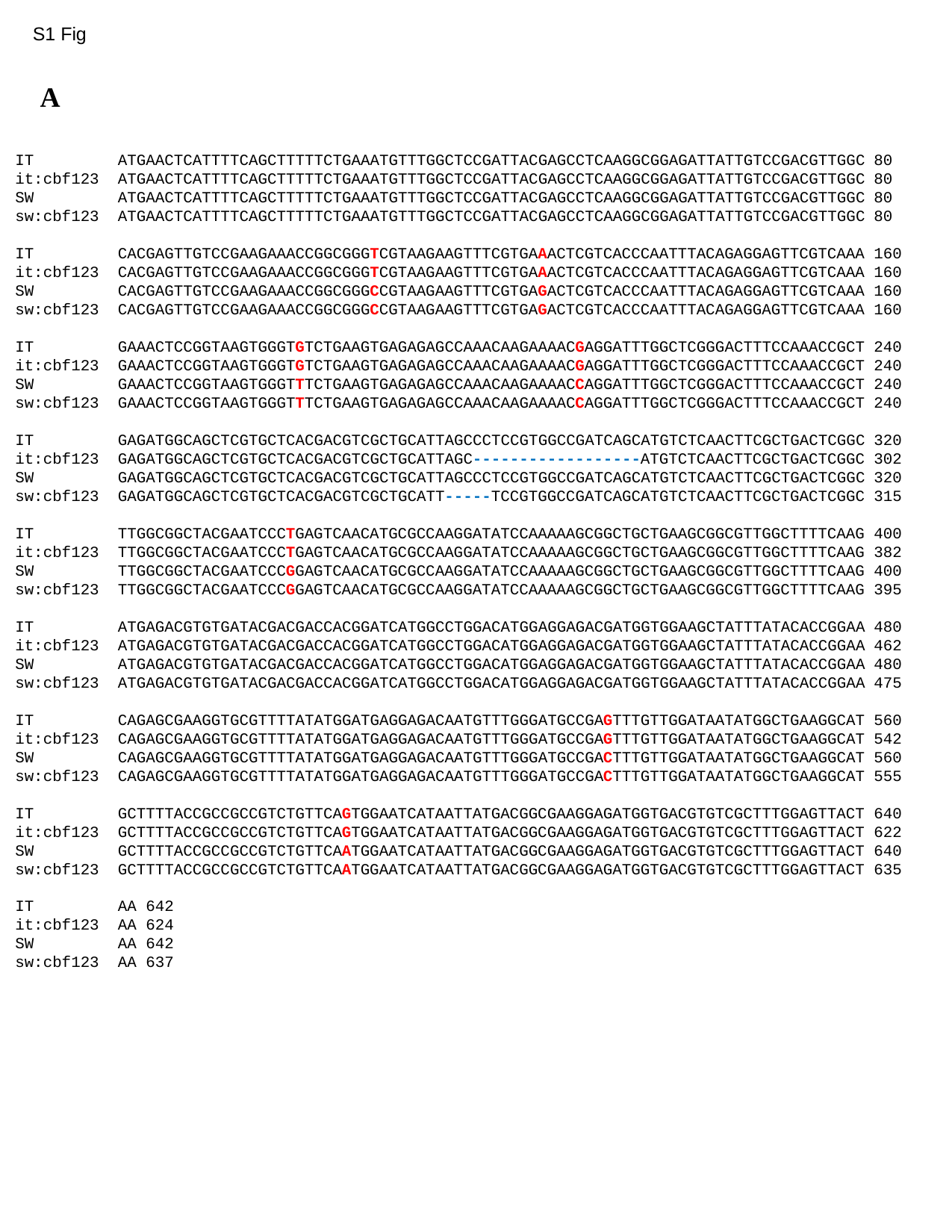

S1 Fig
A
IT ATGAACTCATTTTCAGCTTTTTCTGAAATGTTTGGCTCCGATTACGAGCCTCAAGGCGGAGATTATTGTCCGACGTTGGC 80
it:cbf123 ATGAACTCATTTTCAGCTTTTTCTGAAATGTTTGGCTCCGATTACGAGCCTCAAGGCGGAGATTATTGTCCGACGTTGGC 80
SW ATGAACTCATTTTCAGCTTTTTCTGAAATGTTTGGCTCCGATTACGAGCCTCAAGGCGGAGATTATTGTCCGACGTTGGC 80
sw:cbf123 ATGAACTCATTTTCAGCTTTTTCTGAAATGTTTGGCTCCGATTACGAGCCTCAAGGCGGAGATTATTGTCCGACGTTGGC 80
IT CACGAGTTGTCCGAAGAAACCGGCGGGTCGTAAGAAGTTTCGTGAAACTCGTCACCCAATTTACAGAGGAGTTCGTCAAA 160
it:cbf123 CACGAGTTGTCCGAAGAAACCGGCGGGTCGTAAGAAGTTTCGTGAAACTCGTCACCCAATTTACAGAGGAGTTCGTCAAA 160
SW CACGAGTTGTCCGAAGAAACCGGCGGGCCGTAAGAAGTTTCGTGAGACTCGTCACCCAATTTACAGAGGAGTTCGTCAAA 160
sw:cbf123 CACGAGTTGTCCGAAGAAACCGGCGGGCCGTAAGAAGTTTCGTGAGACTCGTCACCCAATTTACAGAGGAGTTCGTCAAA 160
IT GAAACTCCGGTAAGTGGGTGTCTGAAGTGAGAGAGCCAAACAAGAAAACGAGGATTTGGCTCGGGACTTTCCAAACCGCT 240
it:cbf123 GAAACTCCGGTAAGTGGGTGTCTGAAGTGAGAGAGCCAAACAAGAAAACGAGGATTTGGCTCGGGACTTTCCAAACCGCT 240
SW GAAACTCCGGTAAGTGGGTTTCTGAAGTGAGAGAGCCAAACAAGAAAACCAGGATTTGGCTCGGGACTTTCCAAACCGCT 240
sw:cbf123 GAAACTCCGGTAAGTGGGTTTCTGAAGTGAGAGAGCCAAACAAGAAAACCAGGATTTGGCTCGGGACTTTCCAAACCGCT 240
IT GAGATGGCAGCTCGTGCTCACGACGTCGCTGCATTAGCCCTCCGTGGCCGATCAGCATGTCTCAACTTCGCTGACTCGGC 320
it:cbf123 GAGATGGCAGCTCGTGCTCACGACGTCGCTGCATTAGC------------------ATGTCTCAACTTCGCTGACTCGGC 302
SW GAGATGGCAGCTCGTGCTCACGACGTCGCTGCATTAGCCCTCCGTGGCCGATCAGCATGTCTCAACTTCGCTGACTCGGC 320
sw:cbf123 GAGATGGCAGCTCGTGCTCACGACGTCGCTGCATT-----TCCGTGGCCGATCAGCATGTCTCAACTTCGCTGACTCGGC 315
IT TTGGCGGCTACGAATCCCTGAGTCAACATGCGCCAAGGATATCCAAAAAGCGGCTGCTGAAGCGGCGTTGGCTTTTCAAG 400
it:cbf123 TTGGCGGCTACGAATCCCTGAGTCAACATGCGCCAAGGATATCCAAAAAGCGGCTGCTGAAGCGGCGTTGGCTTTTCAAG 382
SW TTGGCGGCTACGAATCCCGGAGTCAACATGCGCCAAGGATATCCAAAAAGCGGCTGCTGAAGCGGCGTTGGCTTTTCAAG 400
sw:cbf123 TTGGCGGCTACGAATCCCGGAGTCAACATGCGCCAAGGATATCCAAAAAGCGGCTGCTGAAGCGGCGTTGGCTTTTCAAG 395
IT ATGAGACGTGTGATACGACGACCACGGATCATGGCCTGGACATGGAGGAGACGATGGTGGAAGCTATTTATACACCGGAA 480
it:cbf123 ATGAGACGTGTGATACGACGACCACGGATCATGGCCTGGACATGGAGGAGACGATGGTGGAAGCTATTTATACACCGGAA 462
SW ATGAGACGTGTGATACGACGACCACGGATCATGGCCTGGACATGGAGGAGACGATGGTGGAAGCTATTTATACACCGGAA 480
sw:cbf123 ATGAGACGTGTGATACGACGACCACGGATCATGGCCTGGACATGGAGGAGACGATGGTGGAAGCTATTTATACACCGGAA 475
IT CAGAGCGAAGGTGCGTTTTATATGGATGAGGAGACAATGTTTGGGATGCCGAGTTTGTTGGATAATATGGCTGAAGGCAT 560
it:cbf123 CAGAGCGAAGGTGCGTTTTATATGGATGAGGAGACAATGTTTGGGATGCCGAGTTTGTTGGATAATATGGCTGAAGGCAT 542
SW CAGAGCGAAGGTGCGTTTTATATGGATGAGGAGACAATGTTTGGGATGCCGACTTTGTTGGATAATATGGCTGAAGGCAT 560
sw:cbf123 CAGAGCGAAGGTGCGTTTTATATGGATGAGGAGACAATGTTTGGGATGCCGACTTTGTTGGATAATATGGCTGAAGGCAT 555
IT GCTTTTACCGCCGCCGTCTGTTCAGTGGAATCATAATTATGACGGCGAAGGAGATGGTGACGTGTCGCTTTGGAGTTACT 640
it:cbf123 GCTTTTACCGCCGCCGTCTGTTCAGTGGAATCATAATTATGACGGCGAAGGAGATGGTGACGTGTCGCTTTGGAGTTACT 622
SW GCTTTTACCGCCGCCGTCTGTTCAATGGAATCATAATTATGACGGCGAAGGAGATGGTGACGTGTCGCTTTGGAGTTACT 640
sw:cbf123 GCTTTTACCGCCGCCGTCTGTTCAATGGAATCATAATTATGACGGCGAAGGAGATGGTGACGTGTCGCTTTGGAGTTACT 635
IT AA 642
it:cbf123 AA 624
SW AA 642
sw:cbf123 AA 637

## Slide 2
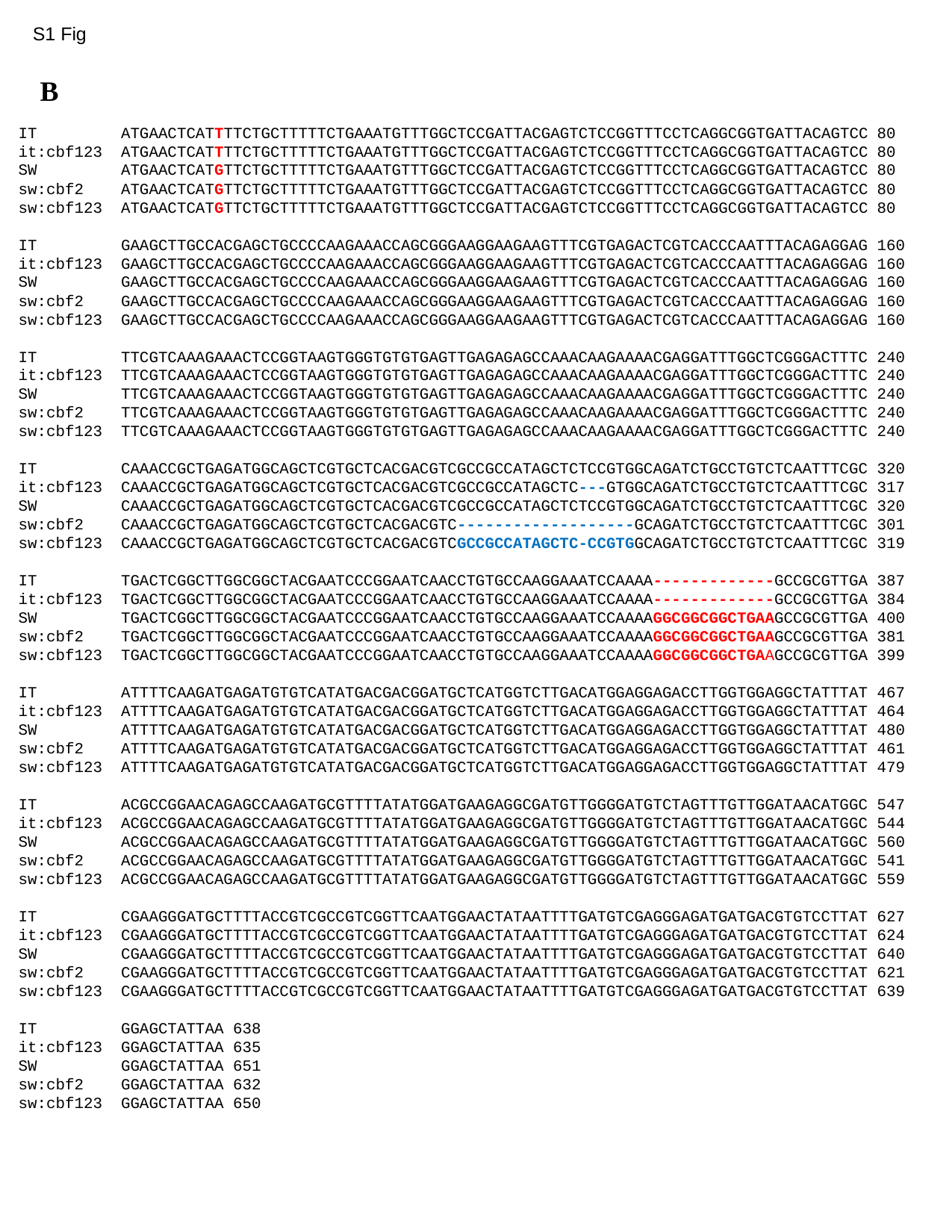

S1 Fig
B
IT ATGAACTCATTTTCTGCTTTTTCTGAAATGTTTGGCTCCGATTACGAGTCTCCGGTTTCCTCAGGCGGTGATTACAGTCC 80
it:cbf123 ATGAACTCATTTTCTGCTTTTTCTGAAATGTTTGGCTCCGATTACGAGTCTCCGGTTTCCTCAGGCGGTGATTACAGTCC 80
SW ATGAACTCATGTTCTGCTTTTTCTGAAATGTTTGGCTCCGATTACGAGTCTCCGGTTTCCTCAGGCGGTGATTACAGTCC 80
sw:cbf2 ATGAACTCATGTTCTGCTTTTTCTGAAATGTTTGGCTCCGATTACGAGTCTCCGGTTTCCTCAGGCGGTGATTACAGTCC 80
sw:cbf123 ATGAACTCATGTTCTGCTTTTTCTGAAATGTTTGGCTCCGATTACGAGTCTCCGGTTTCCTCAGGCGGTGATTACAGTCC 80
IT GAAGCTTGCCACGAGCTGCCCCAAGAAACCAGCGGGAAGGAAGAAGTTTCGTGAGACTCGTCACCCAATTTACAGAGGAG 160
it:cbf123 GAAGCTTGCCACGAGCTGCCCCAAGAAACCAGCGGGAAGGAAGAAGTTTCGTGAGACTCGTCACCCAATTTACAGAGGAG 160
SW GAAGCTTGCCACGAGCTGCCCCAAGAAACCAGCGGGAAGGAAGAAGTTTCGTGAGACTCGTCACCCAATTTACAGAGGAG 160
sw:cbf2 GAAGCTTGCCACGAGCTGCCCCAAGAAACCAGCGGGAAGGAAGAAGTTTCGTGAGACTCGTCACCCAATTTACAGAGGAG 160
sw:cbf123 GAAGCTTGCCACGAGCTGCCCCAAGAAACCAGCGGGAAGGAAGAAGTTTCGTGAGACTCGTCACCCAATTTACAGAGGAG 160
IT TTCGTCAAAGAAACTCCGGTAAGTGGGTGTGTGAGTTGAGAGAGCCAAACAAGAAAACGAGGATTTGGCTCGGGACTTTC 240
it:cbf123 TTCGTCAAAGAAACTCCGGTAAGTGGGTGTGTGAGTTGAGAGAGCCAAACAAGAAAACGAGGATTTGGCTCGGGACTTTC 240
SW TTCGTCAAAGAAACTCCGGTAAGTGGGTGTGTGAGTTGAGAGAGCCAAACAAGAAAACGAGGATTTGGCTCGGGACTTTC 240
sw:cbf2 TTCGTCAAAGAAACTCCGGTAAGTGGGTGTGTGAGTTGAGAGAGCCAAACAAGAAAACGAGGATTTGGCTCGGGACTTTC 240
sw:cbf123 TTCGTCAAAGAAACTCCGGTAAGTGGGTGTGTGAGTTGAGAGAGCCAAACAAGAAAACGAGGATTTGGCTCGGGACTTTC 240
IT CAAACCGCTGAGATGGCAGCTCGTGCTCACGACGTCGCCGCCATAGCTCTCCGTGGCAGATCTGCCTGTCTCAATTTCGC 320
it:cbf123 CAAACCGCTGAGATGGCAGCTCGTGCTCACGACGTCGCCGCCATAGCTC---GTGGCAGATCTGCCTGTCTCAATTTCGC 317
SW CAAACCGCTGAGATGGCAGCTCGTGCTCACGACGTCGCCGCCATAGCTCTCCGTGGCAGATCTGCCTGTCTCAATTTCGC 320
sw:cbf2 CAAACCGCTGAGATGGCAGCTCGTGCTCACGACGTC-------------------GCAGATCTGCCTGTCTCAATTTCGC 301
sw:cbf123 CAAACCGCTGAGATGGCAGCTCGTGCTCACGACGTCGCCGCCATAGCTC-CCGTGGCAGATCTGCCTGTCTCAATTTCGC 319
IT TGACTCGGCTTGGCGGCTACGAATCCCGGAATCAACCTGTGCCAAGGAAATCCAAAA-------------GCCGCGTTGA 387
it:cbf123 TGACTCGGCTTGGCGGCTACGAATCCCGGAATCAACCTGTGCCAAGGAAATCCAAAA-------------GCCGCGTTGA 384
SW TGACTCGGCTTGGCGGCTACGAATCCCGGAATCAACCTGTGCCAAGGAAATCCAAAAGGCGGCGGCTGAAGCCGCGTTGA 400
sw:cbf2 TGACTCGGCTTGGCGGCTACGAATCCCGGAATCAACCTGTGCCAAGGAAATCCAAAAGGCGGCGGCTGAAGCCGCGTTGA 381
sw:cbf123 TGACTCGGCTTGGCGGCTACGAATCCCGGAATCAACCTGTGCCAAGGAAATCCAAAAGGCGGCGGCTGAAGCCGCGTTGA 399
IT ATTTTCAAGATGAGATGTGTCATATGACGACGGATGCTCATGGTCTTGACATGGAGGAGACCTTGGTGGAGGCTATTTAT 467
it:cbf123 ATTTTCAAGATGAGATGTGTCATATGACGACGGATGCTCATGGTCTTGACATGGAGGAGACCTTGGTGGAGGCTATTTAT 464
SW ATTTTCAAGATGAGATGTGTCATATGACGACGGATGCTCATGGTCTTGACATGGAGGAGACCTTGGTGGAGGCTATTTAT 480
sw:cbf2 ATTTTCAAGATGAGATGTGTCATATGACGACGGATGCTCATGGTCTTGACATGGAGGAGACCTTGGTGGAGGCTATTTAT 461
sw:cbf123 ATTTTCAAGATGAGATGTGTCATATGACGACGGATGCTCATGGTCTTGACATGGAGGAGACCTTGGTGGAGGCTATTTAT 479
IT ACGCCGGAACAGAGCCAAGATGCGTTTTATATGGATGAAGAGGCGATGTTGGGGATGTCTAGTTTGTTGGATAACATGGC 547
it:cbf123 ACGCCGGAACAGAGCCAAGATGCGTTTTATATGGATGAAGAGGCGATGTTGGGGATGTCTAGTTTGTTGGATAACATGGC 544
SW ACGCCGGAACAGAGCCAAGATGCGTTTTATATGGATGAAGAGGCGATGTTGGGGATGTCTAGTTTGTTGGATAACATGGC 560
sw:cbf2 ACGCCGGAACAGAGCCAAGATGCGTTTTATATGGATGAAGAGGCGATGTTGGGGATGTCTAGTTTGTTGGATAACATGGC 541
sw:cbf123 ACGCCGGAACAGAGCCAAGATGCGTTTTATATGGATGAAGAGGCGATGTTGGGGATGTCTAGTTTGTTGGATAACATGGC 559
IT CGAAGGGATGCTTTTACCGTCGCCGTCGGTTCAATGGAACTATAATTTTGATGTCGAGGGAGATGATGACGTGTCCTTAT 627
it:cbf123 CGAAGGGATGCTTTTACCGTCGCCGTCGGTTCAATGGAACTATAATTTTGATGTCGAGGGAGATGATGACGTGTCCTTAT 624
SW CGAAGGGATGCTTTTACCGTCGCCGTCGGTTCAATGGAACTATAATTTTGATGTCGAGGGAGATGATGACGTGTCCTTAT 640
sw:cbf2 CGAAGGGATGCTTTTACCGTCGCCGTCGGTTCAATGGAACTATAATTTTGATGTCGAGGGAGATGATGACGTGTCCTTAT 621
sw:cbf123 CGAAGGGATGCTTTTACCGTCGCCGTCGGTTCAATGGAACTATAATTTTGATGTCGAGGGAGATGATGACGTGTCCTTAT 639
IT GGAGCTATTAA 638
it:cbf123 GGAGCTATTAA 635
SW GGAGCTATTAA 651
sw:cbf2 GGAGCTATTAA 632
sw:cbf123 GGAGCTATTAA 650

## Slide 3
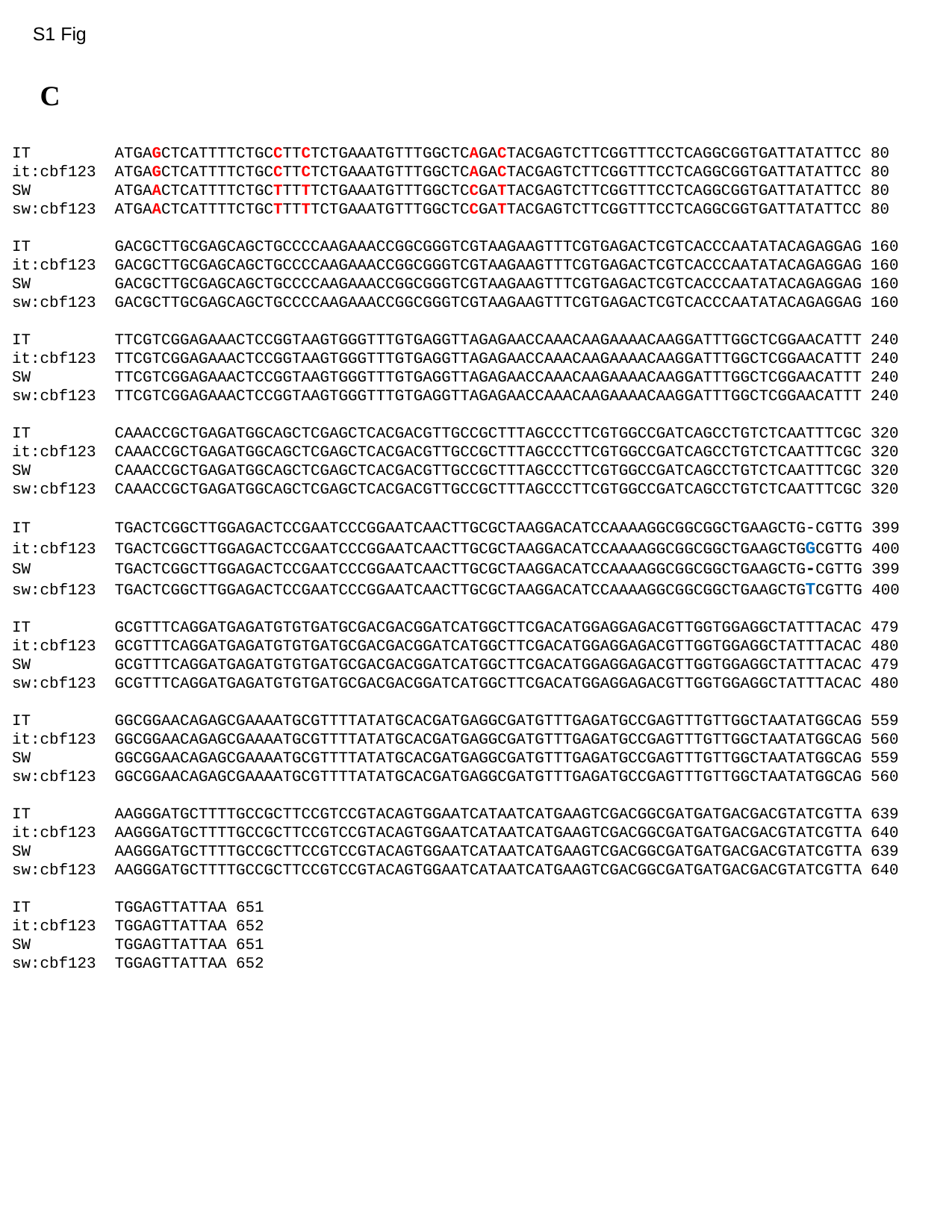

S1 Fig
C
IT ATGAGCTCATTTTCTGCCTTCTCTGAAATGTTTGGCTCAGACTACGAGTCTTCGGTTTCCTCAGGCGGTGATTATATTCC 80
it:cbf123 ATGAGCTCATTTTCTGCCTTCTCTGAAATGTTTGGCTCAGACTACGAGTCTTCGGTTTCCTCAGGCGGTGATTATATTCC 80
SW ATGAACTCATTTTCTGCTTTTTCTGAAATGTTTGGCTCCGATTACGAGTCTTCGGTTTCCTCAGGCGGTGATTATATTCC 80
sw:cbf123 ATGAACTCATTTTCTGCTTTTTCTGAAATGTTTGGCTCCGATTACGAGTCTTCGGTTTCCTCAGGCGGTGATTATATTCC 80
IT GACGCTTGCGAGCAGCTGCCCCAAGAAACCGGCGGGTCGTAAGAAGTTTCGTGAGACTCGTCACCCAATATACAGAGGAG 160
it:cbf123 GACGCTTGCGAGCAGCTGCCCCAAGAAACCGGCGGGTCGTAAGAAGTTTCGTGAGACTCGTCACCCAATATACAGAGGAG 160
SW GACGCTTGCGAGCAGCTGCCCCAAGAAACCGGCGGGTCGTAAGAAGTTTCGTGAGACTCGTCACCCAATATACAGAGGAG 160
sw:cbf123 GACGCTTGCGAGCAGCTGCCCCAAGAAACCGGCGGGTCGTAAGAAGTTTCGTGAGACTCGTCACCCAATATACAGAGGAG 160
IT TTCGTCGGAGAAACTCCGGTAAGTGGGTTTGTGAGGTTAGAGAACCAAACAAGAAAACAAGGATTTGGCTCGGAACATTT 240
it:cbf123 TTCGTCGGAGAAACTCCGGTAAGTGGGTTTGTGAGGTTAGAGAACCAAACAAGAAAACAAGGATTTGGCTCGGAACATTT 240
SW TTCGTCGGAGAAACTCCGGTAAGTGGGTTTGTGAGGTTAGAGAACCAAACAAGAAAACAAGGATTTGGCTCGGAACATTT 240
sw:cbf123 TTCGTCGGAGAAACTCCGGTAAGTGGGTTTGTGAGGTTAGAGAACCAAACAAGAAAACAAGGATTTGGCTCGGAACATTT 240
IT CAAACCGCTGAGATGGCAGCTCGAGCTCACGACGTTGCCGCTTTAGCCCTTCGTGGCCGATCAGCCTGTCTCAATTTCGC 320
it:cbf123 CAAACCGCTGAGATGGCAGCTCGAGCTCACGACGTTGCCGCTTTAGCCCTTCGTGGCCGATCAGCCTGTCTCAATTTCGC 320
SW CAAACCGCTGAGATGGCAGCTCGAGCTCACGACGTTGCCGCTTTAGCCCTTCGTGGCCGATCAGCCTGTCTCAATTTCGC 320
sw:cbf123 CAAACCGCTGAGATGGCAGCTCGAGCTCACGACGTTGCCGCTTTAGCCCTTCGTGGCCGATCAGCCTGTCTCAATTTCGC 320
IT TGACTCGGCTTGGAGACTCCGAATCCCGGAATCAACTTGCGCTAAGGACATCCAAAAGGCGGCGGCTGAAGCTG-CGTTG 399
it:cbf123 TGACTCGGCTTGGAGACTCCGAATCCCGGAATCAACTTGCGCTAAGGACATCCAAAAGGCGGCGGCTGAAGCTGGCGTTG 400
SW TGACTCGGCTTGGAGACTCCGAATCCCGGAATCAACTTGCGCTAAGGACATCCAAAAGGCGGCGGCTGAAGCTG-CGTTG 399
sw:cbf123 TGACTCGGCTTGGAGACTCCGAATCCCGGAATCAACTTGCGCTAAGGACATCCAAAAGGCGGCGGCTGAAGCTGTCGTTG 400
IT GCGTTTCAGGATGAGATGTGTGATGCGACGACGGATCATGGCTTCGACATGGAGGAGACGTTGGTGGAGGCTATTTACAC 479
it:cbf123 GCGTTTCAGGATGAGATGTGTGATGCGACGACGGATCATGGCTTCGACATGGAGGAGACGTTGGTGGAGGCTATTTACAC 480
SW GCGTTTCAGGATGAGATGTGTGATGCGACGACGGATCATGGCTTCGACATGGAGGAGACGTTGGTGGAGGCTATTTACAC 479
sw:cbf123 GCGTTTCAGGATGAGATGTGTGATGCGACGACGGATCATGGCTTCGACATGGAGGAGACGTTGGTGGAGGCTATTTACAC 480
IT GGCGGAACAGAGCGAAAATGCGTTTTATATGCACGATGAGGCGATGTTTGAGATGCCGAGTTTGTTGGCTAATATGGCAG 559
it:cbf123 GGCGGAACAGAGCGAAAATGCGTTTTATATGCACGATGAGGCGATGTTTGAGATGCCGAGTTTGTTGGCTAATATGGCAG 560
SW GGCGGAACAGAGCGAAAATGCGTTTTATATGCACGATGAGGCGATGTTTGAGATGCCGAGTTTGTTGGCTAATATGGCAG 559
sw:cbf123 GGCGGAACAGAGCGAAAATGCGTTTTATATGCACGATGAGGCGATGTTTGAGATGCCGAGTTTGTTGGCTAATATGGCAG 560
IT AAGGGATGCTTTTGCCGCTTCCGTCCGTACAGTGGAATCATAATCATGAAGTCGACGGCGATGATGACGACGTATCGTTA 639
it:cbf123 AAGGGATGCTTTTGCCGCTTCCGTCCGTACAGTGGAATCATAATCATGAAGTCGACGGCGATGATGACGACGTATCGTTA 640
SW AAGGGATGCTTTTGCCGCTTCCGTCCGTACAGTGGAATCATAATCATGAAGTCGACGGCGATGATGACGACGTATCGTTA 639
sw:cbf123 AAGGGATGCTTTTGCCGCTTCCGTCCGTACAGTGGAATCATAATCATGAAGTCGACGGCGATGATGACGACGTATCGTTA 640
IT TGGAGTTATTAA 651
it:cbf123 TGGAGTTATTAA 652
SW TGGAGTTATTAA 651
sw:cbf123 TGGAGTTATTAA 652

## Slide 4
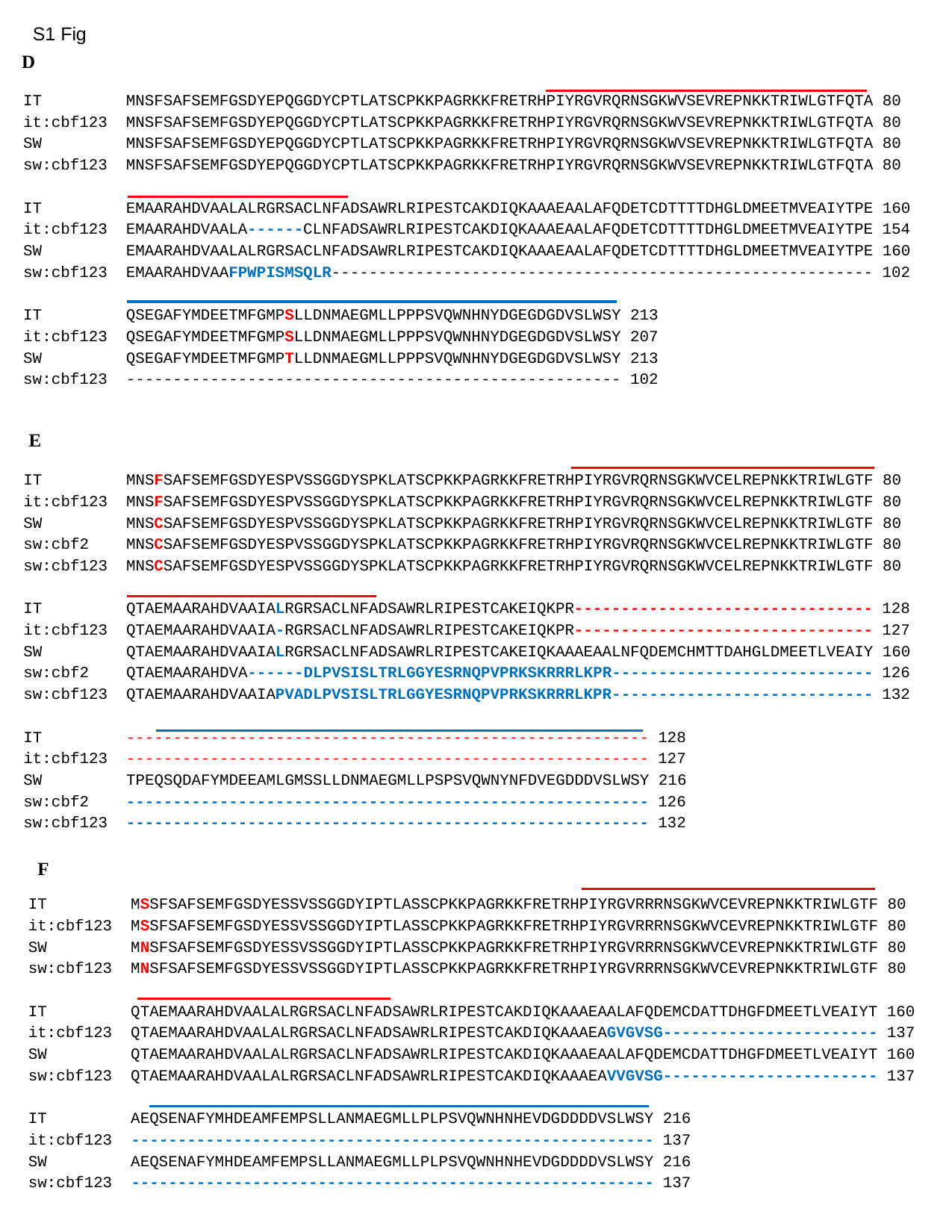

S1 Fig
D
IT MNSFSAFSEMFGSDYEPQGGDYCPTLATSCPKKPAGRKKFRETRHPIYRGVRQRNSGKWVSEVREPNKKTRIWLGTFQTA 80
it:cbf123 MNSFSAFSEMFGSDYEPQGGDYCPTLATSCPKKPAGRKKFRETRHPIYRGVRQRNSGKWVSEVREPNKKTRIWLGTFQTA 80
SW MNSFSAFSEMFGSDYEPQGGDYCPTLATSCPKKPAGRKKFRETRHPIYRGVRQRNSGKWVSEVREPNKKTRIWLGTFQTA 80
sw:cbf123 MNSFSAFSEMFGSDYEPQGGDYCPTLATSCPKKPAGRKKFRETRHPIYRGVRQRNSGKWVSEVREPNKKTRIWLGTFQTA 80
IT EMAARAHDVAALALRGRSACLNFADSAWRLRIPESTCAKDIQKAAAEAALAFQDETCDTTTTDHGLDMEETMVEAIYTPE 160
it:cbf123 EMAARAHDVAALA------CLNFADSAWRLRIPESTCAKDIQKAAAEAALAFQDETCDTTTTDHGLDMEETMVEAIYTPE 154
SW EMAARAHDVAALALRGRSACLNFADSAWRLRIPESTCAKDIQKAAAEAALAFQDETCDTTTTDHGLDMEETMVEAIYTPE 160
sw:cbf123 EMAARAHDVAAFPWPISMSQLR---------------------------------------------------------- 102
IT QSEGAFYMDEETMFGMPSLLDNMAEGMLLPPPSVQWNHNYDGEGDGDVSLWSY 213
it:cbf123 QSEGAFYMDEETMFGMPSLLDNMAEGMLLPPPSVQWNHNYDGEGDGDVSLWSY 207
SW QSEGAFYMDEETMFGMPTLLDNMAEGMLLPPPSVQWNHNYDGEGDGDVSLWSY 213
sw:cbf123 ----------------------------------------------------- 102
E
IT MNSFSAFSEMFGSDYESPVSSGGDYSPKLATSCPKKPAGRKKFRETRHPIYRGVRQRNSGKWVCELREPNKKTRIWLGTF 80
it:cbf123 MNSFSAFSEMFGSDYESPVSSGGDYSPKLATSCPKKPAGRKKFRETRHPIYRGVRQRNSGKWVCELREPNKKTRIWLGTF 80
SW MNSCSAFSEMFGSDYESPVSSGGDYSPKLATSCPKKPAGRKKFRETRHPIYRGVRQRNSGKWVCELREPNKKTRIWLGTF 80
sw:cbf2 MNSCSAFSEMFGSDYESPVSSGGDYSPKLATSCPKKPAGRKKFRETRHPIYRGVRQRNSGKWVCELREPNKKTRIWLGTF 80
sw:cbf123 MNSCSAFSEMFGSDYESPVSSGGDYSPKLATSCPKKPAGRKKFRETRHPIYRGVRQRNSGKWVCELREPNKKTRIWLGTF 80
IT QTAEMAARAHDVAAIALRGRSACLNFADSAWRLRIPESTCAKEIQKPR-------------------------------- 128
it:cbf123 QTAEMAARAHDVAAIA-RGRSACLNFADSAWRLRIPESTCAKEIQKPR-------------------------------- 127
SW QTAEMAARAHDVAAIALRGRSACLNFADSAWRLRIPESTCAKEIQKAAAEAALNFQDEMCHMTTDAHGLDMEETLVEAIY 160
sw:cbf2 QTAEMAARAHDVA------DLPVSISLTRLGGYESRNQPVPRKSKRRRLKPR---------------------------- 126
sw:cbf123 QTAEMAARAHDVAAIAPVADLPVSISLTRLGGYESRNQPVPRKSKRRRLKPR---------------------------- 132
IT -------------------------------------------------------- 128
it:cbf123 -------------------------------------------------------- 127
SW TPEQSQDAFYMDEEAMLGMSSLLDNMAEGMLLPSPSVQWNYNFDVEGDDDVSLWSY 216
sw:cbf2 -------------------------------------------------------- 126
sw:cbf123 -------------------------------------------------------- 132
F
IT MSSFSAFSEMFGSDYESSVSSGGDYIPTLASSCPKKPAGRKKFRETRHPIYRGVRRRNSGKWVCEVREPNKKTRIWLGTF 80
it:cbf123 MSSFSAFSEMFGSDYESSVSSGGDYIPTLASSCPKKPAGRKKFRETRHPIYRGVRRRNSGKWVCEVREPNKKTRIWLGTF 80
SW MNSFSAFSEMFGSDYESSVSSGGDYIPTLASSCPKKPAGRKKFRETRHPIYRGVRRRNSGKWVCEVREPNKKTRIWLGTF 80
sw:cbf123 MNSFSAFSEMFGSDYESSVSSGGDYIPTLASSCPKKPAGRKKFRETRHPIYRGVRRRNSGKWVCEVREPNKKTRIWLGTF 80
IT QTAEMAARAHDVAALALRGRSACLNFADSAWRLRIPESTCAKDIQKAAAEAALAFQDEMCDATTDHGFDMEETLVEAIYT 160
it:cbf123 QTAEMAARAHDVAALALRGRSACLNFADSAWRLRIPESTCAKDIQKAAAEAGVGVSG----------------------- 137
SW QTAEMAARAHDVAALALRGRSACLNFADSAWRLRIPESTCAKDIQKAAAEAALAFQDEMCDATTDHGFDMEETLVEAIYT 160
sw:cbf123 QTAEMAARAHDVAALALRGRSACLNFADSAWRLRIPESTCAKDIQKAAAEAVVGVSG----------------------- 137
IT AEQSENAFYMHDEAMFEMPSLLANMAEGMLLPLPSVQWNHNHEVDGDDDDVSLWSY 216
it:cbf123 -------------------------------------------------------- 137
SW AEQSENAFYMHDEAMFEMPSLLANMAEGMLLPLPSVQWNHNHEVDGDDDDVSLWSY 216
sw:cbf123 -------------------------------------------------------- 137

## Slide 5
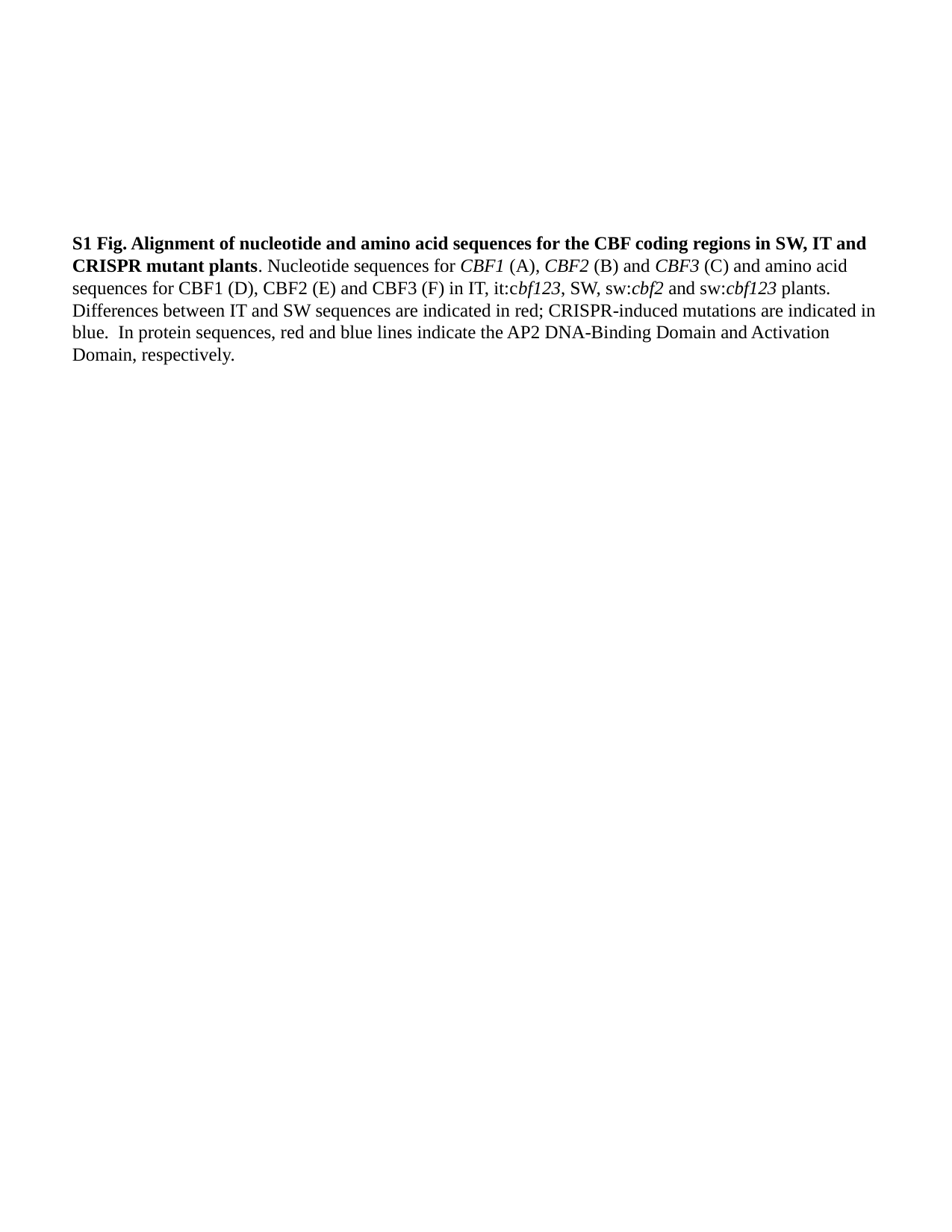

S1 Fig. Alignment of nucleotide and amino acid sequences for the CBF coding regions in SW, IT and CRISPR mutant plants. Nucleotide sequences for CBF1 (A), CBF2 (B) and CBF3 (C) and amino acid sequences for CBF1 (D), CBF2 (E) and CBF3 (F) in IT, it:cbf123, SW, sw:cbf2 and sw:cbf123 plants. Differences between IT and SW sequences are indicated in red; CRISPR-induced mutations are indicated in blue. In protein sequences, red and blue lines indicate the AP2 DNA-Binding Domain and Activation Domain, respectively.
