# Supplementary material for: CBF-dependent and CBF-independent regulatory pathways contribute to the differences in freezing tolerance and cold-regulated gene expression of two Arabidopsis ecotypes locally adapted to sites in Sweden and Italy
Source: PLoS One. 2018 Dec 5;13(12):e0207723. doi: 10.1371/journal.pone.0207723 (PMC6281195; doi:10.1371/journal.pone.0207723)
Supplement: S2 Fig — The cbf1 and cbf3 alleles and corresponding CBF1 and CBF3 WT coding sequences from the IT ecotype were overexpressed in Ws-2 plants to determine whether they could induce expression of CBF regulon genes. (A) Photographs show that overexpression of the cbf1 and cbf3 alleles did not retard plant growth consistent with the proteins being non-functional. (B) Overexpression of cbf1 and cbf3 did not induce expression of the CBF regulon genes Gols3, COR15a or COR47. Names in parentheses on the x-axis indicate the lines from which the overexpressed alleles are cloned. Error bars indicate SE for three biological replicates. (PPTX) [file pone.0207723.s002.pptx]

## Slide 1
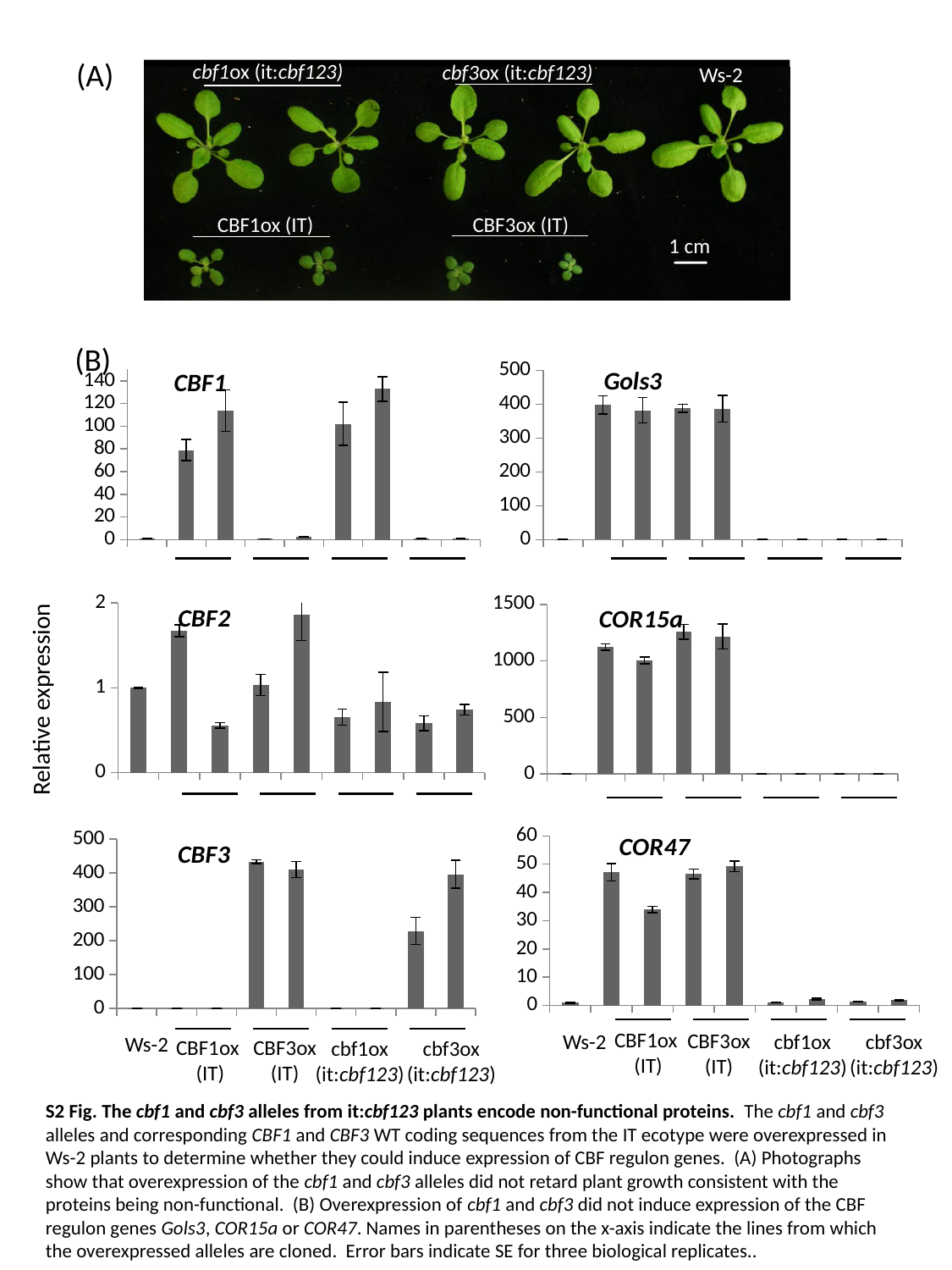

(A)
cbf1ox (it:cbf123)
cbf3ox (it:cbf123)
Ws-2
CBF1ox (IT)
CBF3ox (IT)
1 cm
(B)
### Chart:
| Category | CBF1 |
|---|---|
| Ws-2 | 1.0055608391640494 |
| IT-CBF1ox | 79.01276762302219 |
| IT-CBF1ox | 113.76280736978883 |
| IT-CBF3ox | 0.7736975875770575 |
| IT-CBF3ox | 2.4180654766630063 |
| IT8-CBF1ox | 102.1641015667099 |
| IT8-CBF1ox | 132.75127372153668 |
| IT8-CBF3ox | 0.9977184714282198 |
| IT8-CBF3ox | 1.0261408081256667 |
### Chart: Gols3
| Category | GolS3 |
|---|---|
| Ws-2 | 1.0196543306221806 |
| IT-CBF1ox | 397.5211167582948 |
| IT-CBF1ox | 382.1787960421348 |
| IT-CBF3ox | 388.05819192941044 |
| IT-CBF3ox | 386.64053504941813 |
| IT8-CBF1ox | 0.8935607302011649 |
| IT8-CBF1ox | 1.1282257443702663 |
| IT8-CBF3ox | 0.6675343716870596 |
| IT8-CBF3ox | 0.9655659405684124 |
### Chart: CBF2
| Category | CBF1 |
|---|---|
| Ws-2 | 1.0000198617899048 |
| IT-CBF1ox | 1.6721152358862554 |
| IT-CBF1ox | 0.5566215791677541 |
| IT-CBF3ox | 1.0358732571478357 |
| IT-CBF3ox | 1.859358659997629 |
| IT8-CBF1ox | 0.6546432796064322 |
| IT8-CBF1ox | 0.834806662224435 |
| IT8-CBF3ox | 0.5826189219190825 |
| IT8-CBF3ox | 0.7436432073113233 |
### Chart: COR15a
| Category | GolS3 |
|---|---|
| Ws-2 | 1.0006257622107984 |
| IT-CBF1ox | 1120.9315341993738 |
| IT-CBF1ox | 1002.2878301785662 |
| IT-CBF3ox | 1254.9558260643423 |
| IT-CBF3ox | 1215.1495489066128 |
| IT8-CBF1ox | 0.5905089355208536 |
| IT8-CBF1ox | 0.6703626840204296 |
| IT8-CBF3ox | 0.31757069397313886 |
| IT8-CBF3ox | 0.5691269815294243 |Relative expression
### Chart: COR47
| Category | GolS3 |
|---|---|
| Ws-2 | 1.0203057580893016 |
| IT-CBF1ox | 47.089414095406134 |
| IT-CBF1ox | 33.90618053606428 |
| IT-CBF3ox | 46.53065586948645 |
| IT-CBF3ox | 49.268490359042715 |
| IT8-CBF1ox | 1.1112899990105911 |
| IT8-CBF1ox | 2.221386067945307 |
| IT8-CBF3ox | 1.3218635952966444 |
| IT8-CBF3ox | 1.856060215891106 |
### Chart: CBF3
| Category | CBF1 |
|---|---|
| Ws-2 | 1.0011961111370256 |
| IT-CBF1ox | 0.8332050281392099 |
| IT-CBF1ox | 0.602035854276506 |
| IT-CBF3ox | 432.7787785713627 |
| IT-CBF3ox | 409.4633418430806 |
| IT8-CBF1ox | 0.712556576311372 |
| IT8-CBF1ox | 0.6622877530915054 |
| IT8-CBF3ox | 228.91525749421157 |
| IT8-CBF3ox | 395.8299588172899 |CBF1ox
(IT)
CBF3ox
(IT)
Ws-2
cbf3ox
(it:cbf123)
cbf1ox
(it:cbf123)
Ws-2
CBF1ox
(IT)
CBF3ox
(IT)
cbf1ox
(it:cbf123)
cbf3ox
(it:cbf123)
S2 Fig. The cbf1 and cbf3 alleles from it:cbf123 plants encode non-functional proteins. The cbf1 and cbf3 alleles and corresponding CBF1 and CBF3 WT coding sequences from the IT ecotype were overexpressed in Ws-2 plants to determine whether they could induce expression of CBF regulon genes. (A) Photographs show that overexpression of the cbf1 and cbf3 alleles did not retard plant growth consistent with the proteins being non-functional. (B) Overexpression of cbf1 and cbf3 did not induce expression of the CBF regulon genes Gols3, COR15a or COR47. Names in parentheses on the x-axis indicate the lines from which the overexpressed alleles are cloned. Error bars indicate SE for three biological replicates..
