# Supplementary material for: CBF-dependent and CBF-independent regulatory pathways contribute to the differences in freezing tolerance and cold-regulated gene expression of two Arabidopsis ecotypes locally adapted to sites in Sweden and Italy
Source: PLoS One. 2018 Dec 5;13(12):e0207723. doi: 10.1371/journal.pone.0207723 (PMC6281195; doi:10.1371/journal.pone.0207723)
Supplement: S3 Fig — The cbf2 allele and corresponding CBF2 coding sequence from the SW ecotype were overexpressed in Ws-2 plants to determine whether they could induce expression of CBF regulon genes. (A) Photographs show that overexpression of the cbf2 allele did not retard plant growth consistent with the protein being non-functional. (B) Overexpression of cbf2 did not induce expression of the CBF regulon genes Gols3, COR15a or COR47. Names in parentheses on the x-axis indicate the lines from which the overexpressed alleles are cloned. Error bars indicate SE for three biological replicates. (PPTX) [file pone.0207723.s003.pptx]

## Slide 1
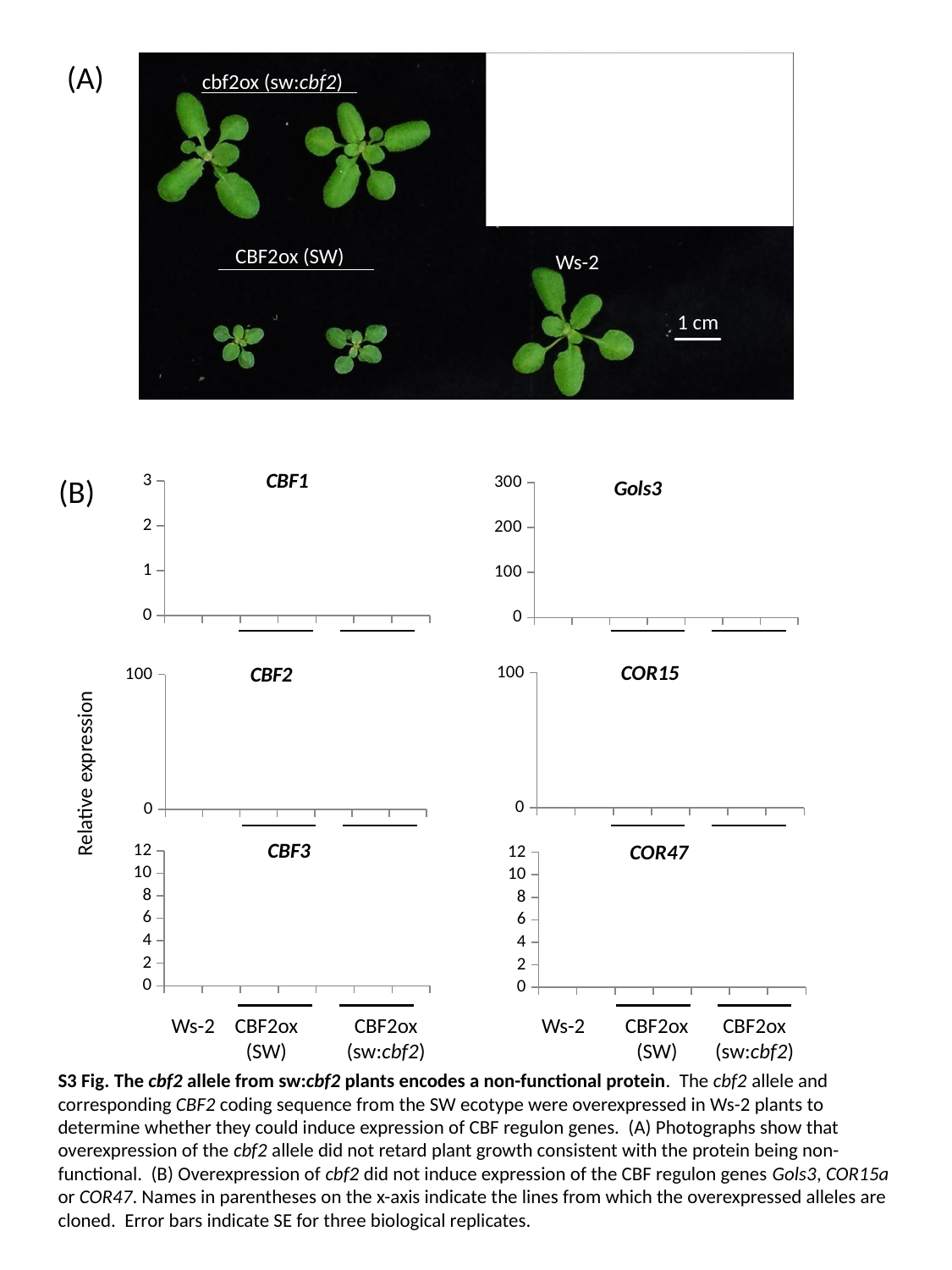

(A)
cbf2ox of sw:cbf23
cbf2ox (sw:cbf2)
CBF2ox (SW)
Ws-2
1 cm
1 cm
### Chart:
| Category | CBF1 |
|---|---|
| Ws2 | 1.1191685759085002 |
| SW:CBF2ox | 1.4022625218143672 |
| SW:CBF2ox | 1.7691243887010761 |
| cbf2ox | 1.613797781818312 |
| cbf2ox | 1.7644895905574718 |
### Chart:
| Category | CBF3 |
|---|---|
| Ws2 | 1.018605314154008 |
| SW-CBF2ox | 170.92922705531126 |
| SW-CBF2ox | 181.35139046418314 |
| sw51-cbf2ox | 2.318007736901864 |
| sw51-cbf2ox | 2.0659935653070836 |(B)
### Chart:
| Category | COR15 |
|---|---|
| Ws2 | 1.1096614294272393 |
| SW-CBF2ox | 356.1177993547303 |
| SW-CBF2ox | 339.88689714632886 |
| sw51-cbf2ox | 5.934796823223366 |
| sw51-cbf2ox | 4.571799877118731 |
### Chart:
| Category | CBF1 |
|---|---|
| Ws2 | 1.0032130082000457 |
| SW-CBF2ox | 149.48272900305452 |
| SW-CBF2ox | 191.96288018476866 |
| sw51-cbf2ox | 190.48677312611085 |
| sw51-cbf2ox | 438.1385396341466 |Relative expression
### Chart:
| Category | CBF3 |
|---|---|
| Ws2 | 1.0371235739410105 |
| SW-CBF2ox | 2.356069128435887 |
| SW-CBF2ox | 2.404189856125269 |
| sw51-cbf2ox | 1.5889552009235306 |
| sw51-cbf2ox | 1.3787431153702265 |
### Chart:
| Category | COR15 |
|---|---|
| Ws2 | 1.0033327317133616 |
| SW-CBF2ox | 10.419054983295219 |
| SW-CBF2ox | 12.586640691347869 |
| sw51-cbf2ox | 1.4054732523207243 |
| sw51-cbf2ox | 1.036474765173163 |Ws-2
CBF2ox
(SW)
CBF2ox
(sw:cbf2)
Ws-2
CBF2ox
(SW)
CBF2ox
(sw:cbf2)
S3 Fig. The cbf2 allele from sw:cbf2 plants encodes a non-functional protein. The cbf2 allele and corresponding CBF2 coding sequence from the SW ecotype were overexpressed in Ws-2 plants to determine whether they could induce expression of CBF regulon genes. (A) Photographs show that overexpression of the cbf2 allele did not retard plant growth consistent with the protein being non-functional. (B) Overexpression of cbf2 did not induce expression of the CBF regulon genes Gols3, COR15a or COR47. Names in parentheses on the x-axis indicate the lines from which the overexpressed alleles are cloned. Error bars indicate SE for three biological replicates.
