# Supplementary material for: CBF-dependent and CBF-independent regulatory pathways contribute to the differences in freezing tolerance and cold-regulated gene expression of two Arabidopsis ecotypes locally adapted to sites in Sweden and Italy
Source: PLoS One. 2018 Dec 5;13(12):e0207723. doi: 10.1371/journal.pone.0207723 (PMC6281195; doi:10.1371/journal.pone.0207723)
Supplement: S4 Fig — (A) Venn diagram showing the number of common and specific CBF regulon genes. Genes were assigned to the CBF regulon if they were induced at least two-fold in response to low temperature (4°C for 24 h) and down-regulated in the cbf123 triple mutant by at least two-fold (log2 = 1, FDR = 0.05). (B, C) The average minimum monthly temperatures for the genotypes were plotted against the number of CBF regulon genes (B) or total number of COR genes (C). A regression line for the data and R2 value are shown. Red indicates it:cbf123; black, col:cbf123; blue, sw:cbf123. (PPTX) [file pone.0207723.s004.pptx]

## Slide 1
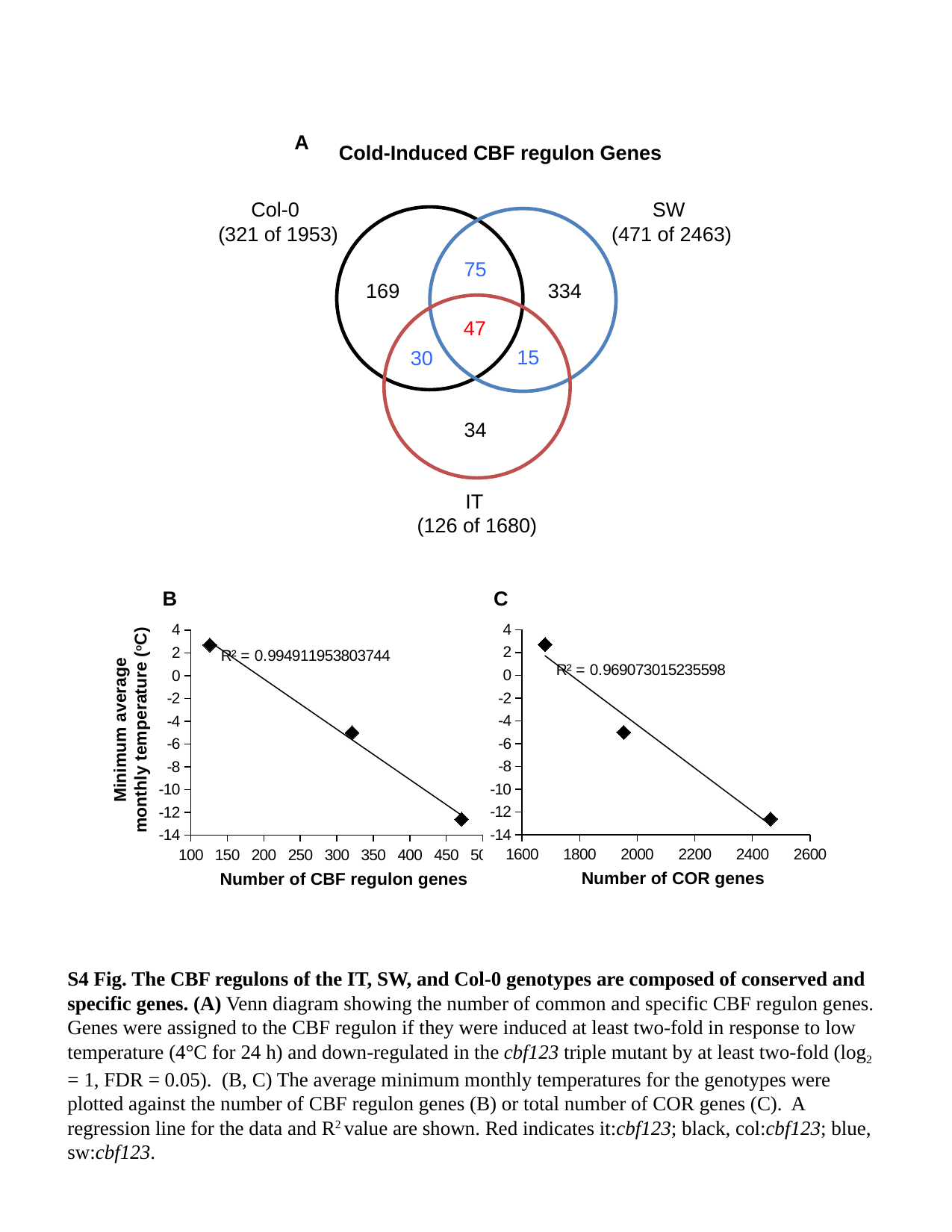

A
Cold-Induced CBF regulon Genes
Col-0
(321 of 1953)
SW
(471 of 2463)
75
169
334
47
15
30
34
IT
(126 of 1680)
B
C
### Chart
| Category | |
|---|---|
### Chart
| Category | |
|---|---|Minimum average monthly temperature (oC)
Number of COR genes
Number of CBF regulon genes
S4 Fig. The CBF regulons of the IT, SW, and Col-0 genotypes are composed of conserved and specific genes. (A) Venn diagram showing the number of common and specific CBF regulon genes. Genes were assigned to the CBF regulon if they were induced at least two-fold in response to low temperature (4°C for 24 h) and down-regulated in the cbf123 triple mutant by at least two-fold (log2 = 1, FDR = 0.05). (B, C) The average minimum monthly temperatures for the genotypes were plotted against the number of CBF regulon genes (B) or total number of COR genes (C). A regression line for the data and R2 value are shown. Red indicates it:cbf123; black, col:cbf123; blue, sw:cbf123.
