# Supplementary material for: CBF-dependent and CBF-independent regulatory pathways contribute to the differences in freezing tolerance and cold-regulated gene expression of two Arabidopsis ecotypes locally adapted to sites in Sweden and Italy
Source: PLoS One. 2018 Dec 5;13(12):e0207723. doi: 10.1371/journal.pone.0207723 (PMC6281195; doi:10.1371/journal.pone.0207723)
Supplement: S1 Table — (DOCX) [file pone.0207723.s005.docx]

**S1 Table. Primers used for quantitative RT-PCR.**

| **Gene** | **Locus** | **For/Rev** | **Primer** |
| --- | --- | --- | --- |
| *CBF1* | AT4G25490 | For | 5’- GGAGACAATGTTTGGGATGC-3’ |
|  |  | Rev | 5’- TTAGTAACTCCAAAGCGACACG-3’ |
| *CBF2* | AT4G25470 | For | 5’- CGACGGATGCTCATGGTCTT-3’ |
|  |  | Rev | 5’- TCTTCATCCATATAAAACGCATCTTG-3’ |
| *CBF3* | AT4G25480 | For | 5’- TTCCGTCCGTACAGTGGAAT-3’ |
|  |  | Rev | 5’- AACTCCATAACGATACGTCGTC-3’ |
| *COR15A* | AT2G42540 | For | 5’- CGTTGATCTACGCCGCTAAAG-3’ |
|  |  | Rev | 5’- TGGCCTCGTTGAGGTCATC-3’ |
| *COR47* | AT1G20440 | For | 5’- CGGTACCAGTGTCGGAGAGT-3’ |
|  |  | Rev | 5’- ACAGCTGGTGAATCCTCTGC-3’ |
| *GOLS3* | AT1G09350 | For | 5’- CTGACGAGCGAGGTTCTTGTC-3’ |
|  |  | Rev | 5’- AACAAATTCTAAGTAAACATCACCAG-3’ |
| *MAF4* | AT5G65070 | For | 5’-CTAAAGTCCCTGGAAGAGCAGC-3’ |
|  |  | Rev | 5’-TCTGGTTCTCCTCTCTCAGCAG-3’ |
| *MAF5* | AT5G65080 | For | 5’-TCCATGGAAGAGCAGCTCAAGA-3’ |
|  |  | Rev | 5’-CACCTCGCTAGCTAGAACCTTG-3’ |
| *FLC* | AT5G10140 | For | 5’-GGCTAGCCAGATGGAGAATAATCA-3’ |
|  |  | Rev | 5’-ATTTCAACCGCCGATTTAAGGTG-3’ |
| *IPP2* | AT3G02780 | For | 5’-TGCTCTTCATCGTGCGAGACGT-3’ |
|  |  | Rev | 5’-GCTCCTTCAGCTCTTCCCGGC-3’ |
