# Supplementary material for: CBF-dependent and CBF-independent regulatory pathways contribute to the differences in freezing tolerance and cold-regulated gene expression of two Arabidopsis ecotypes locally adapted to sites in Sweden and Italy
Source: PLoS One. 2018 Dec 5;13(12):e0207723. doi: 10.1371/journal.pone.0207723 (PMC6281195; doi:10.1371/journal.pone.0207723)
Supplement: S2 Table — EL50 values were calculated from fitted third-order linear polynomial trends from the curves shown in Fig 2 and are shown in the diagonal boxes. Two-way analysis of variance was performed for the electrolyte leakage experiment; significance of comparisons of responses among genotypes was determined by orthogonal contrast. P values representing comparisons of the temperature response curves are indicated in the intersecting cells. ns = not significantly different Comparison of each genotype in both Fig 2A & 2B and in Fig 2C & 2D indicated the curves were very highly significantly different (P<0.0001) between warm and 2 wk cold. (DOCX) [file pone.0207723.s006.docx]

**S2 Table. EL_50_ values and statistical analysis of temperature response curves.**

EL_50_ values were calculated from fitted third-order linear polynomial trends from the curves shown in Fig 2 and are shown in the diagonal boxes. Two-way analysis of variance was performed for the electrolyte leakage experiment; significance of comparisons of responses among genotypes was determined by orthogonal contrast. P values representing comparisons of the temperature response curves are indicated in the intersecting cells.

| **Fig 2A. warm** | | | | |
| --- | --- | --- | --- | --- |
|  | ***sw:cbf123*** | **IT** | ***sw:cbf2*** | **SW** |
| ***sw:cbf123*** | -4.5 | ns | ns | ns |
| **IT** |  | -4.6 | ns | ns |
| ***sw:cbf2*** |  |  | -4.7 | ns |
| **SW** |  |  |  | -5.1 |

| **Fig 2B. 2 wk cold** | | | | |
| --- | --- | --- | --- | --- |
|  | ***sw:cbf123*** | **IT** | ***sw:cbf2*** | **SW** |
| ***sw:cbf123*** | -8.9 | P=0.034 | P<0.0001 | P<0.0001 |
| **IT** |  | -9.6 | P<0.0001 | P<0.0001 |
| ***sw:cbf2*** |  |  | -11.6 | P<0.001 |
| **SW** |  |  |  | -12.4 |

| **Fig 2C. warm** | | |
| --- | --- | --- |
|  | ***it:cbf123*** | **IT** |
| ***it:cbf123*** | -3.8 | P<0.001 |
| **IT** |  | -4.7 |

| **Fig 2D. 2 wk cold** | | |
| --- | --- | --- |
|  | ***it:cbf123*** | **IT** |
| ***it:cbf123*** | -7.3 | P<0.0001 |
| **IT** |  | -8.7 |

ns = not significantly different

Comparison of each genotype in both Fig 2A&B and in Fig 2C&D indicated the curves were very highly significantly different (P<0.0001) between warm and 2 wk cold.
